# Dietary Nutrient Adequacy and Supplement Use in German Community-Dwelling Older Adults Compared to a Group of Younger People: Findings from the Berlin Aging Study II (BASE-II)

**DOI:** 10.3390/nu18183077

**Published:** 2026-09-20

**Authors:** Katharina Heimberg, Annett Martin, Anke Ehlers, Karen Ildico Hirsch-Ernst, Ilja Demuth, Dominik Spira, Elisabeth Steinhagen-Thiessen

**Affiliations:** 1 Department of Food Safety, Federal Institute for Risk Assessment (BfR), 10589 Berlin, Germanykaren-ildico.hirsch-ernst@bfr.bund.de (K.I.H.-E.); 2 Department Exposure, Federal Institute for Risk Assessment (BfR), 10589 Berlin, Germany; 3 Department of Endocrinology and Metabolic Diseases, Charité–Universitätsmedizin Berlin, 13353 Berlin, Germany; ilja.demuth@charite.de (I.D.);; 4 BCRT—Berlin Institute of Health Center for Regenerative Therapies, 10117 Berlin, Germany

**Keywords:** community-dwelling older adults, dietary intake, supplements, risk for inadequate intake, healthy ageing, nutritional assessment, (Estimated) Average Requirement (EAR), Average Requirement (AR), Berlin Aging Study II (BASE-II)

## Abstract

**Background:** As populations age, evaluating nutrient intake in older adults becomes increasingly important. While the nutritional status of nursing home residents and hospitalised older patients has been widely studied, less is known about community-dwelling older adults. This study assessed dietary nutrient and supplement intake in the Berlin Aging Study II (BASE-II). **Methods:** Nutrient and supplement intake was assessed in 1671 community-dwelling BASE-II participants aged 60–84 years using the EPIC Potsdam FFQ. Supplement use was additionally recorded during physician interviews. Intakes were compared with reference values of the German Nutrition Society (DGE). The proportion of participants with potentially inadequate nutrient intake was estimated using the (E)AR Cut-Point Method and a probabilistic approach for iron. Data from 500 participants aged 22–36 years served as a comparison group. **Results:** Mean intakes met DGE reference values for most micronutrients in both age groups, whereas fat and protein intake exceeded recommendations. The proportion at risk of inadequate calcium intake was higher among older than younger participants (34 vs. 26%), while younger participants more frequently had inadequate intakes of several B vitamins, particularly vitamin B1 (25 vs. 22%), vitamin B12 (3.7 vs. 0.6%), vitamin B2 (32 vs. 26%), vitamin B6 (30 vs. 20%) and vitamin B9 (folate, 64 vs. 55%). Additionally, more than 15% of participants in both groups were at risk of inadequate intakes of magnesium and vitamin D. For iron, the probabilistic approach indicated a risk of inadequate intake among 37% of younger women. Supplement use was more common among older participants (47.3% vs. 43.0%), although the difference was not statistically significant. **Conclusions:** In this relatively healthy, community-dwelling BASE-II population, older adults did not generally have less favourable nutrient intakes than younger participants. However, the higher proportion at risk of inadequate calcium intake warrants attention. Given the specific characteristics of the BASE-II cohort, these findings cannot be assumed to represent the wider German older population.

## 1. Introduction

Living a long and healthy life is a goal that is embraced by people worldwide [1]. In Europe, similar to other industrialised regions, there is an ongoing change in population demographics, with a growing number of people aged 65 years and older, rising from 90.5 million in 2019 to reach an expected 129.8 million by 2050 [2]. Nevertheless, although ageing is often considered to be associated with frailty and disability [3], the older population—in contrast to younger age groups—is characterised by a marked heterogeneity, which has its causes both in an individually different process of ageing and in the number and severity of individual comorbidities [4]. Ageing is accompanied by physiological changes that may affect dietary intake and nutrient bioavailability. Undernutrition or malnutrition in older adults, for instance, is complex and may be related to factors such as a progressive decline in appetite and food intake, commonly referred to as the anorexia of ageing, age-related changes in taste and smell, reduced enjoyment/skill in meal preparation and consumption, as well as changes in the types and amounts of foods consumed. Furthermore, ageing may be associated with changes in gastrointestinal function, including alterations in gastric secretion, changes in the gut microbiota, or changes in the skin’s synthesis of vitamin D due to possible reduced sun exposure or the skin’s altered production capacity. In addition, renal impairment, which becomes more prevalent with increasing age, may reduce the conversion of vitamin D to its active form, 1,25-dihydroxyvitamin D, thereby contributing to altered vitamin D metabolism in older adults. This altogether may affect the metabolism, absorption or bioavailability of certain nutrients. Chronic diseases and nutrient interactions with medications are additional parameters possibly leading to undernutrition or malnutrition in older people. [3,5,6,7,8,9,10,11]. Therefore, assessing the proportion of a group or population that is at risk of nutrient inadequacy is an important public health and policy concern [4] since nutrient deficiencies may have an impact on the development or progression of chronic illnesses [1] and on functional parameters in older adults, such as frailty or difficulties with independent living [3], which in turn is associated with increased economic and health costs. While there are numerous data on older residents of nursing homes or hospitalised older patients in Germany which have shown that up to a quarter of this population is affected by malnutrition and nutrient deficiencies [12], only a few studies have been conducted with predominantly healthy older people. For instance, the GISELA study [13] and the representative German National Nutrition Survey II (Nationale Verzehrsstudie II, NVS II), which included participants aged 14 to 80 years [14,15]. Thus, the present analysis aimed to evaluate nutrient intake in the Berlin Aging Study II (BASE-II) study population, which comprises older people living independently at home, in comparison to a group of younger people in order to assess whether older, generally healthy, people may have a higher risk of inadequate intakes than younger people.

Dietary supplement use is common among older adults. Taken as supplements to complement the usual diet, they may contribute to improving nutrient intake in some cases. However, dietary supplements are legally considered food in the EU and are not intended to cure or prevent diseases [16]. For these cases, medicinal products with vitamins, minerals or other substances that have been studied in terms of effectiveness and safety profile are available. Furthermore, medicinal products contain mostly single substances as opposed to a combination of various substances as often seen in food supplements. These differences may not be very clear when considering taking vitamins, minerals or other substances. Furthermore, the potential benefits of supplementation need to be considered alongside possible risks since excessive intake of certain vitamins and minerals, particularly when multiple products are consumed concurrently or products that contain various substances, may result in intakes above established Tolerable Upper Intake Levels (ULs). Moreover, supplements may contribute to polypharmacy and may interact with prescribed medications or other supplements. These issues are of particular relevance in older adults because of the high prevalence of multimorbidity and medication use [17,18,19]. Since data on the regulatory status of supplements taken by adults, and supplement use in general, are scarce, the present analysis aimed to provide insight into this topic.

## 2. Methods

### 2.1. Study Population and Design

BASE-II is a multidisciplinary cohort study, which comprised 2200 adult volunteers from the Berlin metropolitan area, with baseline assessments being conducted between 2009 and 2014. The study aimed at the identification and characterisation of factors associated with “healthy” vs. “unhealthy” ageing and included parameters related to geriatrics, internal medicine, immunology, genetics, psychology, sociology and economics. Potential participants were drawn from a pool of individuals originally recruited at the Max Planck Institute for Human Development as part of a number of earlier projects with a focus on neurocognition, and participant recruitment for these and other studies was based on advertisements in local newspapers and the public commuter transport system [20]. For BASE-II, older individuals aged around 60 to 80 years who were living independently and functioning well were recruited. Those who had trouble walking 0.4 km (a quarter of a mile) without assistance, had Parkinson’s disease, a history of stroke or myocardial infarction, had undergone head, heart, or vascular surgery, or suffered from dementia or malignant disease were excluded at the time of recruitment [21]. Baseline characteristics that were assessed during the anamnesis included—among various others—BMI, weight-to-height ratio, physical activity, smoking, blood pressure, alcohol consumption, subjective health and education. Additional information on the methods/devices used can be found elsewhere [20,21].

A group of younger adults between the ages of 20 and 36 years was also recruited and included as a reference group to provide a descriptive reference for nutrient intake. [20]. For the current analysis, cross-sectional data were available for 2171 predominantly healthy participants, of whom about 75% were aged 60 to 84 years and 25% aged 22 to 36 years. The two age groups were not matched and differed in several characteristics. Furthermore, as the data that were analysed in the present study were obtained at a single point in time and the study design is cross-sectional, it is not possible to establish temporal relationships or infer causality between age, dietary intake, and supplement use.

Since the study involved only participants from Berlin, the results may not be considered representative for the German population.

All participants provided written informed consent, and the study was conducted in accordance with the Declaration of Helsinki and approved by the Ethics Committee of the Charité Universitätsmedizin Berlin—approval number EA2/029/09.

Further detailed information about the cohort profile can be found elsewhere [20].

### 2.2. Collection of Nutrient and Supplement Intake Data

For the assessment of the usual nutrient intake (only from food, not from supplements) during the past 12 months, the validated self-administered EPIC-Food Frequency Questionnaire (FFQ) was handed out to the participants (EPIC: European Prospective Investigation into Cancer and Nutrition; Potsdam, Germany). Details on the validity and reproducibility of the questionnaire have been published elsewhere [22,23,24]. Besides 148 single food items, the FFQ included additional questions on specific aspects, such as fat content of dairy products and types of fat used for food preparation [25]. The amount of each food item consumed and the respective nutrient intake per day were calculated using the German Federal Food Code version 3.01 (Bundeslebensmittelschlüssel, BLS 3.01), which is a food and nutrient database developed specifically for epidemiologic studies [26]. Participants showing an implausibly high or low energy intake (below 800 and above 6200 kcal/day, similar to the EPIC Potsdam study with intakes below 800 and above 6000 kcal) were excluded (n = 2). The intake data are reported as intakes via the usual diet without supplements.

It should be noted that the iodine intake data are without consideration of iodine from iodised salt and that the BLS only provides data for vitamin K1; thus, an intake of vitamin K2 is not reported. Regarding vitamin A, the BLS provided information on retinol equivalents (RE), retinol and β-carotene, but not on retinol activity equivalents (RAEs). Vitamin A intake was therefore assessed using the available retinol equivalent (RE) values. For comparison with the Tolerable Upper Intake Level (UL), the approximate intake of retinol and retinyl esters was additionally calculated. As no reference values were available for these individual variables, their absolute intake values are reported in Table S1.

Information on supplement use was obtained from two sources: a detailed medical anamnesis, which recorded the use of pharmaceutical and non-pharmaceutical supplements, and the FFQ, which included additional questions on supplement use. The reference periods differed between the two sources: in the preceding 12 months for the medical anamnesis and at least 1 month within the preceding 2 years for the FFQ. Participants were classified as supplement users if supplement use was reported in either the FFQ or the medical interview. The supplements were classified in the following main groups according to their specific legal regulation within the European Union (EU): medicinal products (MPs)—here further differentiated into MPs with vitamins and/or minerals (MP_VM_), MPs with herbal or other substances (MP_HO_), and homoeopathic MP (hMP)—food for special medical purposes (FSMP) and food supplements (FSs). Missing information was obtained through an internet search. FSMPs include products for people with a medically related nutrient requirement that cannot be met with the intake of normal foods, but are not supposed to cure a disease like an MP. The intake of FS was evaluated in two different ways: FS that could be clearly identified as such by the product label and FS combining the clearly identifiable FS with the ones without further details, which belonged most likely to this category because of their ingredients and which were not labelled as MP (e.g., “chlorella algae” or “protein concentrate”). Multi-supplements were either defined as any of the above-mentioned supplements with ≥5 substances or the participants’ self-reported use of a “multi supplement”. The data from the two separate sources—medical anamnesis and FFQ—were merged and thoroughly checked for duplicate entries and plausibility.

### 2.3. Assessment of Adequacy or Risk for Inadequacy of Nutrient Intake

Reference values for nutrient intake differ between scientific and regulatory bodies, including the German Nutrition Society (DGE), the European Food Safety Authority (EFSA), and the Institute of Medicine/National Academies (IOM/NAM). Although these recommendations are broadly based on similar concepts of nutrient requirements, differences may occur with regard to the underlying evidence, population groups, age categories, and derivation methods. In the present study, the reference values (RVs) of the DGE were used because the study population consisted of participants living in Germany and the DGE recommendations are specifically developed for the German-speaking population. For the assessment of inadequate nutrient intake, the Average Requirement (AR) by the European scientific body EFSA or Estimated Average Requirement (EAR) by IOM/NAM—in case an AR was not available—was used, applying the so-called (E)AR Cut-Point Method. The (E)AR Cut-Point Method allows an estimation of the proportion of the population at increased risk (between 50% and 100% risk) for inadequate nutrient intakes, but does not provide individual risk assessments per person. Since, for iron in younger women, the conditions for applying the (E)AR Cut-Point Method were not met, a probabilistic approach was used to calculate the risk of inadequate intake.

As health risks may be associated both with too low and too high intakes of nutrients, the intake data were also compared to the Tolerable Upper Intake Levels (ULs) derived by EFSA, which correspond to the maximum level of total chronic intake of a particular nutrient from all sources that is judged to be unlikely to pose a risk of adverse health effects in humans [27].

The results are shown as the distribution of the data in box plots and reported as the median attainment of the reference values compared to the respective DGE reference value. The analyses resulting from the use of the (E)AR Cut-Point Method and from the comparison to the ULs are shown as the proportion of the population under/above the respective Value. For further details, see Document S1.

### 2.4. Statistical Analysis

Statistical analysis was carried out using the software package SPSS Statistics version 26 and the statistical software R version 4.0.2. Regarding general participant characteristics, differences between the age groups were examined using the Mann–Whitney U test and the Chi-square test, whereas differences in terms of nutrient intake were examined using the Mann–Whitney U test or the Kruskal–Wallis test (including a Bonferroni correction) for metric variables, depending on the number of groups. Supplement intake was analysed descriptively with the corresponding 95% confidence intervals (95% CI) for the intake frequency (%).

For the calculation of the risk for inadequate iron intake in younger women, the probability approach according to the National Research Council (NRC) [28,29] was applied, dividing iron losses into two components: basal losses through skin, urine and faeces (excreted iron and unabsorbed iron) and losses during menstruation. The former IOM recommends a lognormal distribution model that fits the data reasonably well to model the prevalence of iron inadequacy. Furthermore, in this probability approach, a value of 0.87 mg/day is assumed for basal losses, which slightly overestimates the actual requirement. Expressed in natural logarithms, the distribution of menstrual iron losses can be described with a mean of 0.81 mg/day and a standard deviation of 0.84 mg/day. The probability of the observed intake not being sufficient to cover the iron losses is calculated by an algorithm that describes the cumulative area under the normal distribution curve to the right of Z (Z score): Z = Ln [(OI × UL_a_) − 0.87] − (−0.81)/0.84, with the available iron = OI × UL_a_ (OI = observed intake (mean intake for the frequency interval) and UL_a_ = upper limit of absorption being 18% or 10% for premenopausal women). The assumed basal loss of iron is represented by 0.87 mg/day, whereas 0.81 stands for the mean value of the logarithmic distribution of menstrual iron losses and 0.84 is the standard deviation of this distribution [29]. Following the calculation of the Z-score in our study, the probability of the standard normal distribution was computed with the function “pnorm” in the statistics software R. This way, the area to the right of Z is determined. This area represents the probability of inadequate intake for different iron intake intervals. The total percentage of the population at risk for iron inadequacy is then obtained by multiplying the probability of inadequacy by the proportion of the population in each intake interval and the subsequent summation of those single percentages of each intake interval [28].

## 3. Results

### 3.1. Characteristics of the Participants

The mean age in the younger group was 29 ± 3 years (median 29; interquartile range 27–31), whereas the mean age in the older group was 69 ± 4 years (median 69; interquartile range 66–71) (Table 1). There were no differences between the groups regarding the proportion of women and men (by study design), but the older age group had a higher Body-Mass Index (BMI), waist-to-height ratio (WHtR), and blood pressure (all *p* < 0.001). The percentage of participants with a BMI above 25 or 30 kg/m^2^ was also higher in the older group than in the younger group (64 vs. 26% or 19 vs. 6%, respectively, *p* < 0.001). In contrast, the younger group had a slightly, but statistically significant, higher number of education years and reported more frequently that they were currently smoking (*p* < 0.001). In both groups, about 90% of the participants reported alcohol consumption, and only a low proportion reported being seldom or never physically active (9% in both groups). When being asked about how they would assess their own health, the younger participants reported a very good health status more frequently, whereas the older group answered more often that their health status was fair (*p* < 0.001). Only a small proportion of people reported having a poor or very poor health status in both groups (Table 1).

### 3.2. Nutrient Intake via the Usual Diet

#### 3.2.1. Macronutrients

**Figure 1:**
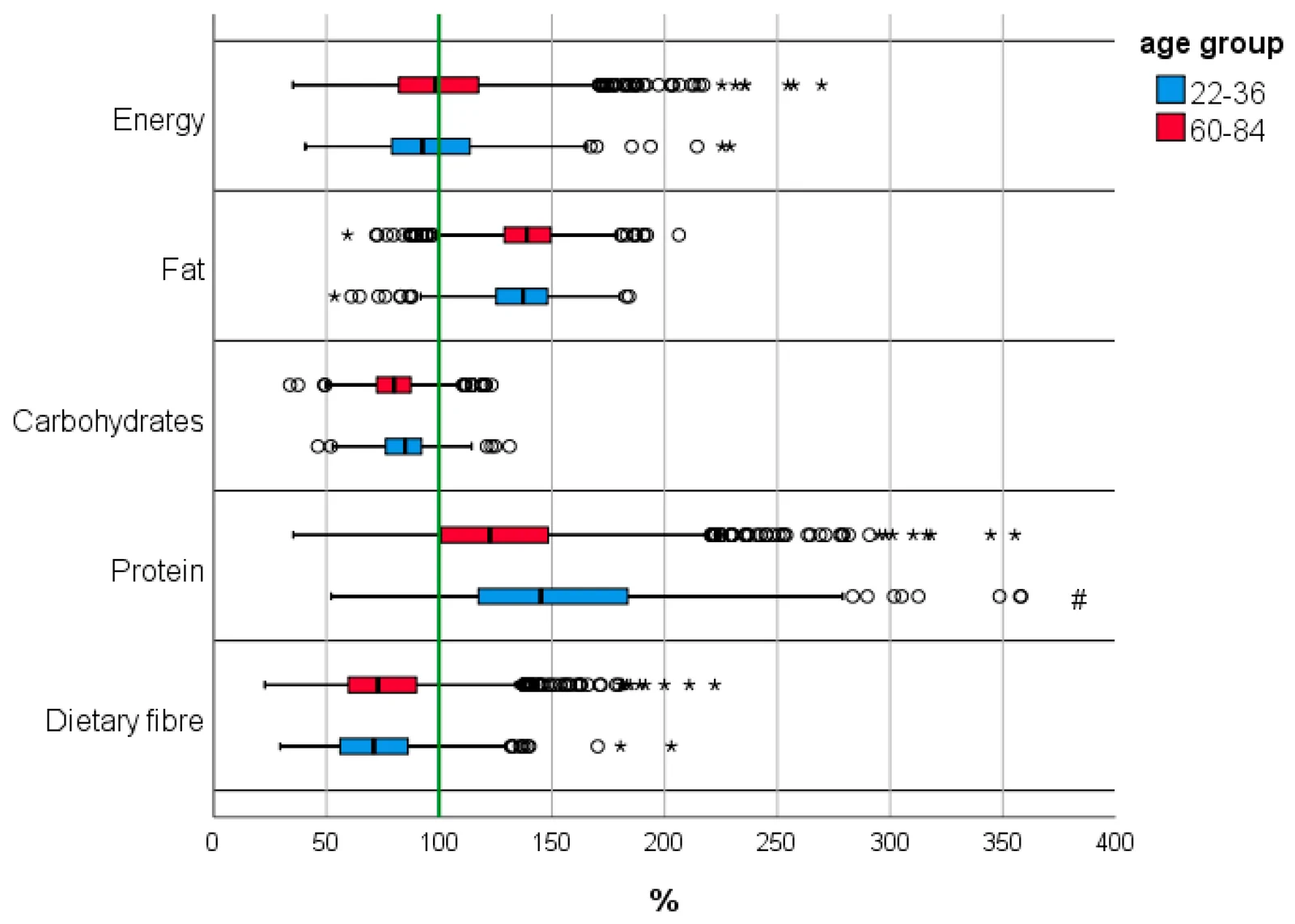
Distribution of macronutrient intake as attainment of the RV (DGE) in %. #: one participant reached 438%. The circles represent outliers (values between 1.5 and 3 times the interquartile range [IQR] beyond the quartiles), where-as the asterisks (*) indicate extreme values (values more than 3 times the IQR beyond the quartiles).

Assuming an average physical activity level, the energy intake (median attainment of the RV) was in line with the RV in both age groups (Figure 1, Table S1), with the older participants reaching the respective RV slightly more often than the younger ones (median attainment of the RV of 98% vs. 93%) (Table S1). Nevertheless, a relatively high proportion of the participants in both age groups had an energy consumption above the RV (Figure 1, Table S1). Regarding fat, besides the median RV attainment in both age groups being about 140%, nearly all of the participants in both age groups consumed more than recommended (the maximum fat intake should not exceed 30% of total energy intake (En%)). There was only a very slight difference in fat intake between the age groups (Figure 1, Table S1). For carbohydrates and protein, the difference between the groups was clearer, with the younger participants reaching the RVs (more than 50 En% and 47 to 67 g per day, respectively) significantly more often (85% vs. 80% and 145% vs. 122% attainment of the carbohydrate and protein RVs, respectively) (Figure 1, Table S1). Regarding protein, in contrast to fat and carbohydrates, an AR and a recommendation for maximum intake (two-fold the RV, both derived by EFSA) were available for comparison. Only a few participants had a protein intake below the AR or above the recommendation for maximum intake, except younger men—27% of this group had intakes that were higher than twice the RV (Table S2). Furthermore, a high percentage of participants in both age groups did not reach the RV for dietary fibre intake (Figure 1, Table S2), which is at least 30 g per day. There was only a very slight difference between the age groups (older group 73% vs. younger group 71% of the dietary fibre RV, *p* = 0.012).

With respect to fatty acids, almost all participants in both age groups had a saturated fatty acid (SFA) intake that was too high compared to the RV for maximum intake according to the DGE (7 to 10 En%, Table S2). In contrast, the median polyunsaturated fatty acid (PUFA) intake was below the RV that is recommended by the DGE (up to a max. of 10 En%) in both age groups. The DGE recommends replacing SFA with PUFA up to the RV in the diet. There was only a slight difference between the age groups, with the older participants having a slightly higher attainment of the PUFA RV than the younger ones (67% vs. 64%) (Table S1). A considerable difference between the groups was seen with the intake of the omega-3 PUFAs eicosapentaenoic acid (EPA) and docosahexaenoic acid (DHA). Here, the older participants had a much higher attainment of the RV than the younger ones (Tables S1 and S2).

Concerning participants’ salt intake, besides the median intake being below the recommendations (max. 6 g/day) in both age groups, a substantial proportion of participants had intakes > 6 g, and this was particularly evident for men (61 and 55% of the younger/older men) (Tables S1 and S2). Furthermore, as mentioned before, nearly the whole study group (about 90%) reported the consumption of alcohol. The median alcohol intake corresponded to 57% of the recommended maximum intake in older participants and 47% in younger participants. Nevertheless, a substantial proportion of participants exceeded the recommended maximum intake, with 44% of older and 38% of younger participants exceeding the respective recommendations (Tables S1 and S2). Details regarding sex differences in macronutrient intakes are shown in Tables S1 and S2.

#### 3.2.2. Minerals and Trace Elements

**Figure 2:**
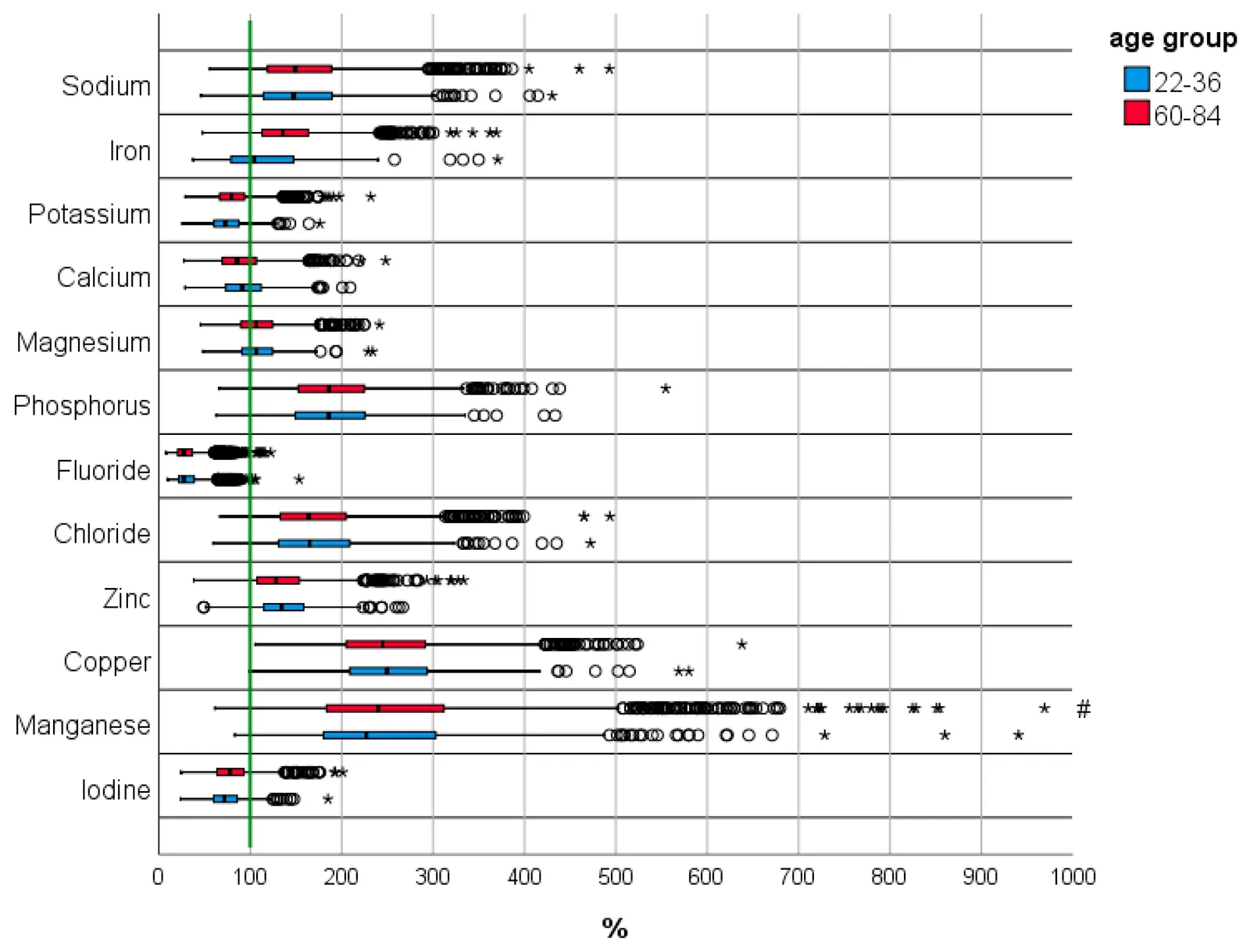
Distribution of mineral intake as attainment of the RV (DGE) in %. #: two participants reached 1102 and 1193%. The circles represent outliers (values between 1.5 and 3 times the interquartile range [IQR] beyond the quartiles), where-as the asterisks (*) indicate extreme values (values more than 3 times the IQR beyond the quartiles).

The median attainment of the RV was either in line with or (well) above the DGE recommendations for the majority of the minerals and trace elements (sodium, magnesium, phosphorus, chloride, zinc, copper, and manganese) in both age groups. A very high median attainment was observed for manganese and copper. In contrast, a median attainment below the RV was observed for potassium, calcium, and iodine in both age groups (Figure 2, Table S1). Notably, with zinc and copper, 0.9 and 2.0% of the younger men vs. 0.8 and 1.0 of the older men, respectively, had intakes above the corresponding Tolerable Upper Intake Level (UL) with their usual diet (Table S3). None of the participants reached the UL concerning the other minerals and trace elements. Details regarding sex differences in mineral and trace element intakes are shown in Table S1.

#### 3.2.3. Vitamins

**Figure 3:**
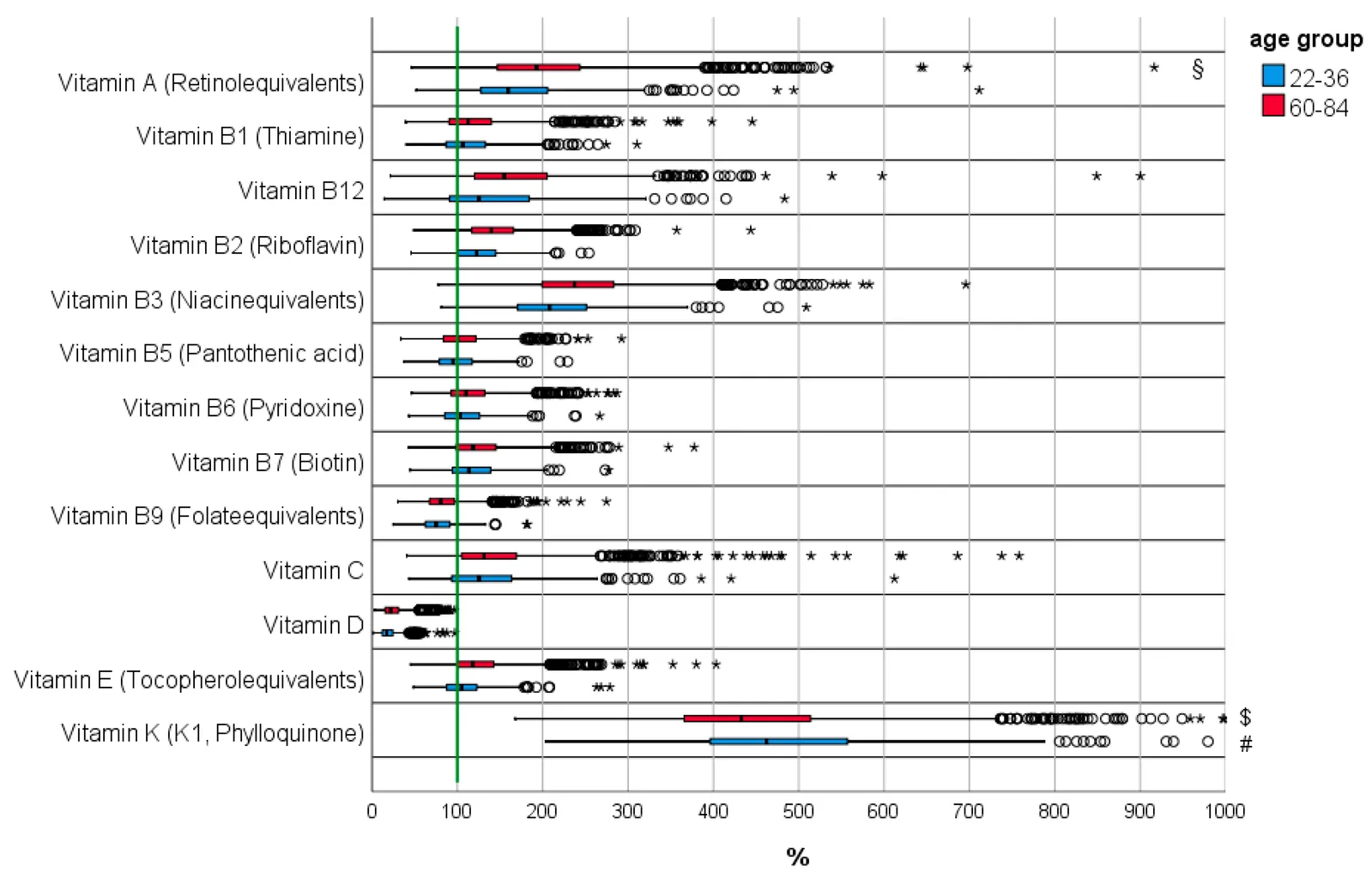
Distribution of vitamin intake as attainment of the RV (DGE) in %. #: two participants reached 1125 and 1136%; §: one participant reached 1364%; $: 14 participants reached more than 1000% (1004, 1022, 1045, 1049, 1062, 1087, 1141, 1143, 1170, 1182, 1383, 1414, 1527, 1540%). The circles represent outliers (values between 1.5 and 3 times the interquartile range [IQR] beyond the quartiles), where-as the asterisks (*) indicate extreme values (values more than 3 times the IQR beyond the quartiles).

Similar to the minerals and trace elements, the median attainment of the RV was in line with or above the recommendations for most of the vitamins in both age groups (total vitamin A expressed as retinol equivalents, vitamin B12, vitamin B2 (riboflavin), vitamin B3 (expressed as niacin equivalents), vitamin B7 (biotin), vitamin C, vitamin E and vitamin K1 (phylloquinone, data for vitamin K2/menaquinones were not available)). A high intake above the recommendations was particularly evident for total vitamin A, vitamin B3 and vitamin K1. In contrast, the RVs for vitamin B9 (folate, expressed as folate equivalents) and vitamin D were not reached by either age group, whereas for vitamin B5 (pantothenic acid), the RV attainment was slightly below the RV in the younger group. Opposed to the minerals and trace elements, where no clear pattern regarding higher attainments of the RVs in one of the age groups was observed, for nearly all of the vitamins (except vitamin K1) a slightly higher attainment of the RVs was observed in the older participants (Figure 3, Table S1) (*p* < 0.01).

Regarding vitamin A, other variables than retinol equivalents were available (retinol and ß-carotene, but not the currently (published by DGE in 2020) used retinol activity equivalents (RAE)). Furthermore, for comparison purposes with the UL, the approximate sum of retinol/retinylesters was calculated. Since there are no RVs for these variables available, the absolute values are reported in Table S1.

For nearly all of the vitamins, the UL was not exceeded with a nutrient intake via the usual diet, except for vitamin A (as the sum of retinol/retinylesters)—2.6% and 3.3% of the younger and older men, as well as 1.6% of the older women had intakes above the UL. Regarding high vitamin A intakes in older women, EFSA noted that the UL in this group does not ensure a sufficient margin of safety in terms of a potential reduction in bone density and risk of fractures [27], but did not derive a separate UL. Younger women did not exceed the UL for vitamin A with their usual diet. Details regarding sex differences in vitamin intakes are shown in the Supplements (Table S1).

### 3.3. Risk for an Inadequate Micronutrient Intake (% of the Population < AR/EAR)

**Figure 4:**
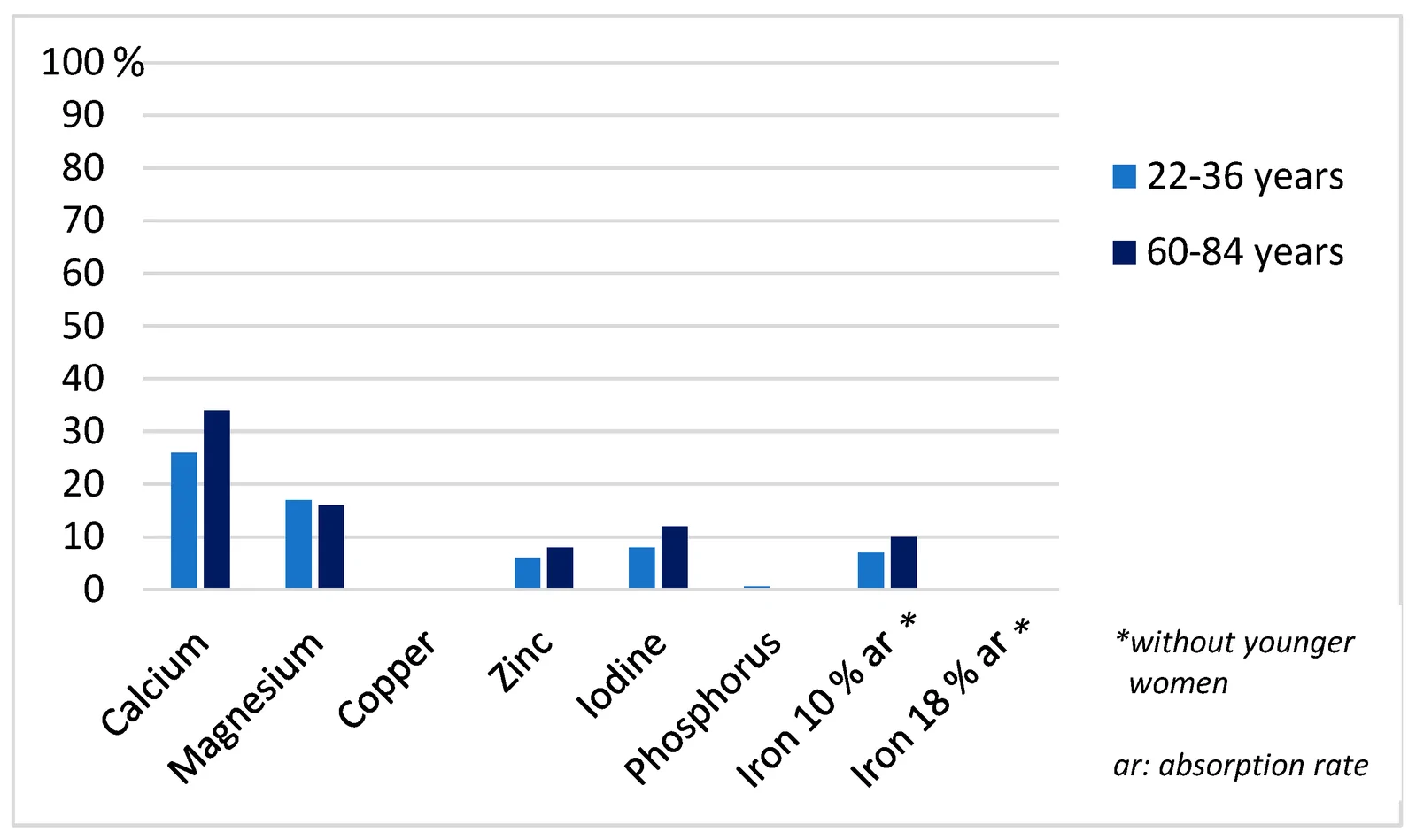
Percentage of participants with an intake of minerals/trace elements below the respective (E)AR. vitamin B1: thiamine, vitamin B2: riboflavin, vitamin B3: niacin equivalents, vitamin B6: pyridoxine, vitamin B9: folate equivalents.

**Figure 5:**
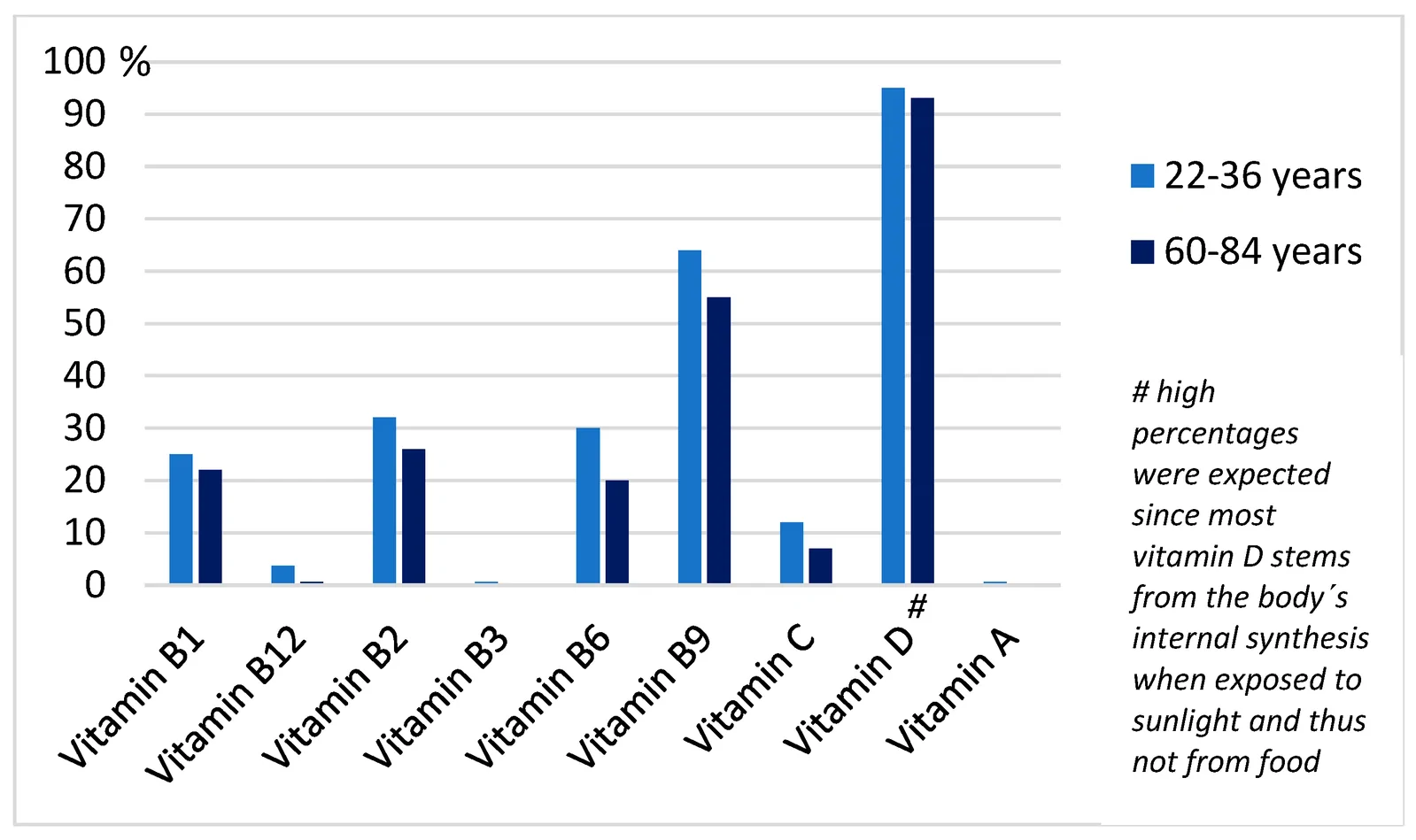
Percentage of participants with an intake of vitamins below the respective (E)AR.

In Figure 4 and Figure 5, and Table S4, the percentage of the respective age group with micronutrient intakes below the respective (E)AR, and thus a higher risk for an inadequate intake, is shown. Regarding minerals and trace elements, a quite high percentage was only observed for calcium and magnesium. Additionally, a high percentage of the younger women had a risk for inadequate iron intake (Table 2). Applying the probability approach for iron, it was shown that the prevalence of inadequate intake was higher than calculated with the (E)AR-Cut-Point Method (Table 2). Considering all the minerals and trace elements, there was no clear pattern of higher nutrient inadequacy in one of the age groups, but a trend to a higher risk for inadequacy in the older participants. A substantial difference between the age groups was only seen for calcium, with the older group having a higher proportion of participants with an intake below the AR. Yet, when considering sex differences, more women than men tended to have a risk for inadequate intakes (Table S4).

In terms of the risk for inadequate vitamin intake, the proportion with an intake below the (E)AR was quite high for vitamin B1 (thiamine), B2 (riboflavin), B6 (pyridoxine), B9 (folate, particularly younger women), and vitamin D (Figure 4, Table S4). In contrast to minerals and trace elements, there was a clear age and gender pattern because, for all of the vitamins, the younger group had a higher percentage of participants with an intake below the (E)AR than the older group, and the same was observed for women vs. men (Figure 5, Table S4). In fact, younger women had the highest percentages with an intake below the (E)AR in nearly all of the vitamins (Table S4).

For an overview of the highest percentages of participants with intakes < (E)AR/RV and > UL/RV regarding macronutrient and micronutrient intake, see Table S5.

### 3.4. Supplement Intake

Nearly half of the study population took supplements, with a prevalence of 47.3% in the older group vs. 43.0% in the younger group. Food supplements were taken most frequently in both groups. It has to be noted that a considerable part of the supplements could not be classified into any of the supplement categories since there was not enough information available (Table 3). Regarding a difference in supplement intake between the two age groups (split by sex), the older participants took supplements more frequently in all of the supplement categories than the younger group (except in the multi-supplement category), but the difference was not significant (*p* > 0.05). When comparing gender groups, older women had the highest supplement intake of all participants and younger men the lowest in nearly all of the supplement categories (except FS that included the ones that were assumed to be FS due to the ingredients, and multi-supplements, in which older men had the lowest intake) (Table 3).

## 4. Discussion

### 4.1. Macronutrient Intake

In this study, macro- and micronutrient intake as well as supplement use among participants of the BASE-II cohort were examined. As in all dietary assessment studies, nutrient intake estimation is subject to uncertainties. In particular, underreporting of energy and nutrient intake is a recognised limitation of self-reported dietary assessment and may result from difficulties in recalling portion sizes, omissions of foods or beverages, or the underreporting of foods perceived as unhealthy. Conversely, overreporting may also occur [12,22,30,31]. Furthermore, this instrument does not capture short-term dietary variation or day-to-day variability in intake with the same precision as repeated dietary records or 24 h recalls. Nevertheless, FFQs are particularly suited to epidemiological studies because they allow the assessment of habitual dietary intake in large populations [32]. The EPIC-FFQ used here has been extensively validated, yet previous analyses indeed indicate underreporting of energy intake, particularly among individuals with high energy consumption [22,30,31] and among women [12]. In the present study, implausible high or low values were excluded; thus, only participants with energy intakes within a predefined plausible range (872 and 6184 kcal) were included, similar to the EPIC Germany approach [33]. In addition, energy intakes were compared to BMI data and assessed for plausibility. However, the Goldberg method to identify potential underreporters was not applied. Because nutrient intake is strongly correlated with energy intake [34,35], general underreporting of nutrients in the present analysis cannot be ruled out and remains uncertain. Potential underreporting may have led to an underestimation of habitual energy and nutrient intake and may consequently have increased the estimated proportion of participants with nutrient intakes below the EAR. However, the magnitude and direction of this potential bias are likely to vary between individuals and nutrients and cannot be determined from the available data.

According to the German Nutrition Society (DGE), at least 50% of daily energy intake should come from carbohydrates and less than 30% from fat [36]. In both age groups, the observed macronutrient pattern was unfavourable: carbohydrate and dietary fibre intake were too low, while fat intake was too high. Protein intake exceeded recommended values substantially. Differences between age groups were modest regarding intakes of total fat, PUFA and dietary fibre, with the older group having slightly higher values, whereas the differences in carbohydrate and protein intake were clearer, with younger participants consuming more of these macronutrients. This pattern mirrors findings from the representative German National Nutrition Survey II (NVS II) [14,15], which was conducted with two different methods (a 24 h recall method and a diet history approach); thus, the results differ slightly.

Higher dietary fibre intake is associated with protective effects against cardiovascular disease, type 2 diabetes, obesity, elevated total and LDL cholesterol, hypertension, and certain cancers [37,38,39]. Since fibre is found almost exclusively in plant-based foods, increasing the consumption of whole grains, legumes, fruits, vegetables, nuts, and seeds may improve intake.

Fat quality is also important. The DGE recommends limiting saturated fatty acids (SFA) to 7–10 En% and increasing PUFA intake up to 10 En% with regard to a lower risk of coronary heart disease [36]. In our study, nearly all participants exceeded the SFA recommendation, while PUFA intake was insufficient. Similar results were reported in NVS II [15]. With regard to the long-chain omega-3 fatty acids EPA and DHA, DGE does not give a specific recommendation, but EFSA considers 250 mg EPA + DHA per day adequate for reducing cardiovascular risk [40]. Intake of EPA and DHA was higher among older participants. Younger women in our study frequently failed to reach the RV, perhaps reflecting lower fish consumption, although food group intake data were not available to confirm this. Improving fat quality may be achieved by reducing consumption of animal-based foods and increasing intake of plant-based foods and vegetable oils such as canola and walnut oil. EPA and DHA are found almost exclusively in marine fish such as mackerel, salmon, herring or tuna [36].

Median protein intake was well above recommended values. Although EFSA considers intakes up to twice the recommended value safe for healthy adults [41], a substantial proportion of younger men (27%) exceeded this threshold, and 5% additionally took protein supplements at an unknown dosage. Because long-term effects of very high protein intake remain uncertain, reducing intake may be advisable for individuals with extremely high consumption. The EPIC-FFQ validation suggests potential underreporting of protein intake [30], but whether this applies to the BASE-II population was not determined.

The slightly higher intake of favourable nutrients like dietary fibre, PUFA and EPA + DHA in the older study group could be attributed to changes in eating behaviour and food preferences (which could also be due to physiological and metabolic changes), greater awareness of healthy eating, consumption of foods with a higher nutrient density, or factors like available time for food preparation.

### 4.2. Micronutrient Intake

Most minerals, trace elements, and vitamins were consumed at or well above recommended levels. The very high manganese intake observed is attributable to calculations based on the lower DGE reference value; when considering the full recommended range, median intake falls within acceptable limits. However, potassium, calcium, iodine, folate and vitamin D intakes did not meet recommendations in any age group, and vitamin B5 (pantothenic acid) intake was insufficient among younger participants. These findings are broadly consistent with NVS II.

No clear age-related pattern emerged for minerals and trace elements, but older participants met vitamin reference values more frequently. This picture was different in NVS II, with younger participants reaching the recommended values more frequently (diet history) or no clear age-related pattern either (24 h recall). The differences between our results and NVS II may stem from methodological differences (FFQ vs. Diet history and 24 h recall; presentation of absolute nutrient values in NVS II vs. relative values in BASE-II), different versions of the German Federal Food Code for nutrient contents in foods (BLS 3.01 vs. 3.02), and differences in population characteristics.

For vitamin K, the assessment was limited to vitamin K1 (phylloquinone), as information on vitamin K2 (menaquinones) was not available in the BLS database used for the calculation of nutrient intake. Consequently, the estimated vitamin K intake does not represent total vitamin K intake, and the contribution of vitamin K2 from dietary sources could not be assessed. Vitamin K1 (phylloquinone) showed the highest relative reference value attainment, but these results are subject to considerable uncertainty. German studies report widely varying vitamin K intakes [42,43,44,45,46,47], likely due to differences in survey methods and nutrient databases. EFSA highlights substantial uncertainties in vitamin K intake estimates, particularly for vitamin K2 (menaquinones) [48]. Without detailed food group consumption and dietary pattern data, assessing the plausibility of high vitamin K1 intakes is difficult, although plant-rich diets may contribute to elevated values. Overall, further research on vitamin K food contents and intake, particularly K2, in Germany and Europe is needed in order to precisely assess vitamin K1, vitamin K2 and total vitamin K intake.

Similar factors as for the observed slightly higher intake of some favourable macronutrients in the older group could be responsible for the slightly higher vitamin and iron intake in the older group.

### 4.3. Risk of Inadequate Nutrient Supply

High prevalences of intake below the (E)AR were observed for calcium, magnesium, folate, vitamin D, and some B vitamins (B1, B2, and B6), and for iron among younger women. A prolonged (severe) inadequate intake of calcium/vitamin D, folate, or iron, for example, may lead to poorer bone health or osteoporosis, pancytopenia (hematologic disorder), or different forms of anaemia [49,50,51].

The (E)AR Cut-Point Method estimates the proportion of the population at increased risk (between 50% and 100%) for inadequate nutrient intakes, but does not provide individual risk assessments [52]. Low vitamin D intake via food was expected, as few foods, such as fish, crustaceans, eggs, milk, and other dairy products, contain substantial amounts [14], and endogenous synthesis is the primary source (80–90%). It is noteworthy that the RVs according to DGE and the (E)AR by the former IOM and EFSA have been derived for a situation in which none or very little vitamin D is synthesised endogenously [15,42,53,54]. Results on vitamin D status of the BASE-II population, assessed using 25(OH)-vitamin D serum levels, have been published elsewhere [55].

Folate intake was also frequently inadequate, particularly among younger women. Intake data alone do not allow a reliable assessment of folate status. Biomarker data from the representative DEGS I study indicate a generally adequate folate status in German adults, but not when applying the higher WHO thresholds for women of childbearing age (non-adequate supply in about 95% of those women) [56,57]. Consequently, folic acid supplementation is recommended for women planning pregnancy in Germany to prevent neural tube defects in their children.

Interestingly, the risk of inadequate iodine intake was not pronounced in our study, despite the BLS not accounting for iodised salt use at home or in processed foods. Thus, the actual intake may be slightly higher than calculated if considering the intake from iodised salt at home and from processed foods. However, the use of iodised salt by the food industry in processed foods in Germany is declining and currently amounts to only about 29% [58]. These points should be considered when interpreting the results for iodine intake. More recent representative biomarker data from the German DEGS II study, using iodine urine excretion, indicate that the iodine supply of the German population might be improved and that parts of the population do not have an adequate iodine status [59]. Iodine is an essential component of thyroid hormones and exerts its effects on a number of metabolic processes in the human body. Iodine deficiency caused by long-lasting inadequate intakes can thus lead to a wide spectrum of pathophysiologic conditions including reduced cognitive abilities or poorer growth, whereas the most obvious clinical sign of iodine deficiency is goitre [59]. The differing results in our study may reflect methodological variation or specific dietary habits, such as consumption of algae products.

For potassium and vitamin B5, (E)AR values are not available, preventing risk assessment. The high proportion below the potassium RV by the DGE is plausible given that the DGE doubled the recommended intake in 2016. Increasing consumption of potassium-rich foods such as fruits, vegetables, potatoes, and nuts may improve intake [60].

Only a few studies have been conducted to date using the (E)AR Cut-Point Method for the assessment of the risk for nutrient inadequacy. So far, most of the existing studies have compared intake data with recommended dietary allowances (RDAs), but this commonly overestimates the risk of inadequate intakes [61,62]. In four more recent European studies, (E)ARs were used [63,64,65,66], but the methodological approach was different, i.e., calculating data on the basis of the average nutrient intakes and not on the basis of raw data, thus assuming a normal intake distribution [63] or, from today’s point of view, outdated nutrient data were used [64]. In all of these studies, instead of EFSA ARs, the EARs from the United Kingdom (UK) and from the Nordic Nutrition Recommendations (NNR) as well as the EARs from the former IOM were used to calculate nutrient inadequacy. Since these RVs partly deviate from the EFSA ARs, the comparability of the results is limited.

Similar to our results, high prevalences of inadequate vitamin D, folate, and calcium intake were reported in the aforementioned study results for the German population [63]. High prevalences were also found for iodine [63,64], iron in younger women [64], as well as B2 and potassium in women [64]. Data for magnesium, phosphorus and B vitamins were often lacking or not directly comparable.

In one further study [3], pooled data for Europe, the United States of America, Australia and New Zealand were analysed, but the results are not comparable to our study results due to the data pooling approach.

Regarding the usage of EFSA ARs for the assessment of the risk for nutrient inadequacy, just one study with a focus on estimation of global nutrient intakes [67] was found. Although Passarelli et al. [67] also evaluated data for Germany, these originate from a regional study with children (DONALD study) and are thus not suitable for comparison with our adult population.

Iron inadequacy among younger women was assessed using a probabilistic approach due to the asymmetric distribution of iron requirements. Iron absorption depends on dietary factors such as the form of iron (haem or non-haem iron), inhibitory or beneficial food components [68]; thus, actual absorption rates are uncertain. EFSA assumes 18% absorption for mixed diets [67], but vegetarian diets—reported by some young women—may result in lower absorption. Without food group intake data, this could not be evaluated further. Regarding a probabilistic approach in assessing iron inadequacy in younger women, only two publications were available [66,67], but without data for Germany. Mitsopoulou et al. [66] applied the same approach for their Greek population as in our study, using the Z-score for the calculation of the probability, but with a higher iron absorption of 20% for a diet rich in animal foods. The results showed that 2.1–2.4% of the younger females had a risk of inadequate iron intake, which was lower than in our study. According to Passarelli et al. [67], who also applied the probability method suggested by the NRC, 8 to 23% of the females from European countries (Denmark, Italy, Sweden, Netherlands, Belgium, and Portugal) had a risk of inadequate iron intake, assuming an absorption rate of 16%. There were no publications available for the comparison of our data assuming a moderate iron absorption rate of 10% when consuming a diet high in phytate, as may be seen in vegetarian diets.

### 4.4. Supplement Use

Although nutrient intake should rather be covered with the usual diet via foods, supplement use may help improve the intake of specific nutrients when dietary intake alone is insufficient, particularly in individuals with an increased risk of inadequate intake. However, supplement use does not necessarily imply that an inadequate dietary intake has been corrected, as this depends on the nutrient, dose, duration of use, and individual nutritional status. Supplement use was relatively common in both age groups in the present study, as approximately half of the participants reported supplement use (when taking into account all types of supplements, including medications), similar to other German studies [69,70,71]. NVS II reported a lower prevalence (24.3–27.6% depending on the survey method) [14,15], likely due to differences in assessment methods. BASE-II combined data from medical anamnesis (12-month period) and FFQ (use for at least one month in the past two years), resulting in broader coverage.

As in other studies [14,15,17,69], older individuals and women were more likely to use supplements. Yet, those other studies did not differentiate supplement categories according to legal classification, thus limiting comparability. In BASE-II, food supplements (FS) were most frequently consumed (with only a slight difference between age groups). However, although supplement use was assessed using information from the medical anamnesis and the FFQ, the available information was not sufficient to classify all reported products unambiguously according to the applicable EU regulatory categories. Consequently, the proportion of supplements classified as medicinal products versus food supplements may be underestimated or uncertain. In addition, misclassification of supplement category may have occurred due to a change in composition or marketing of the supplements between the time of data collection and data analysis. A further limitation is that supplement use was assessed using two sources with different reference periods. Although combining the FFQ and medical anamnesis allowed information on supplement use to be captured more comprehensively, the different observation windows may have introduced heterogeneity in the classification of supplement use. In particular, participants may have reported supplement use in one source but not the other because their use had changed over time. Nevertheless, report of supplement use in either one of the assessments was counted altogether as “supplement use”. This may have resulted in some misclassification of actual current supplement use. On the other hand, a reported supplement use in only one of the data sources (and not in both) may be attributed to the different natures of the data collection—the FFQ is a self-administered questionnaire, whereas the medical anamnesis was performed during a detailed interview by a trained medical staff member. This should be considered when interpreting the estimated prevalence of supplement use.

Legally, FS are considered food and must not have pharmacological effects, but are only intended to supplement the normal diet of healthy individuals. They are not intended to prevent or cure diseases, which is assigned to medicinal products taken under medical supervision only; unlike medicinal products, they are not subject to authorisation procedures, and no binding maximum nutrient levels at EU or national level exist for FS. However, it has been shown that the motif of preventing or alleviating medical complaints is important for consumers when buying food supplements. High nutrient intakes from supplements without medical supervision may therefore pose potential health risks. It is important to note that nutrient intake from dietary supplements was not quantitatively integrated with nutrients from dietary intake in the present study. The analysis was intentionally focused on nutrient intake from food, while supplement use was assessed separately. Consequently, total nutrient intake from food and supplements could not be determined, and the assessment of the Tolerable Upper Intake Levels (ULs) reflects dietary intake from food sources only. Therefore, our findings cannot exclude the possibility that some participants exceeded the UL when supplemental intake is taken into account. A separate analysis integrating dietary and supplemental nutrient intake is planned for a subsequent publication.

### 4.5. Strengths and Limitations

This study provides valuable insights into nutrient and supplement intake among predominantly healthy older adults, a population group for which data are scarce. Strengths include the variety of nutrients analysed, a large sample size, a detailed assessment of the risk of inadequate intake using the (E)AR Cut-Point Method and probabilistic approach, and the use of a validated FFQ and comprehensive nutrient database (BLS). Supplement data were thoroughly evaluated, classified, and analysed for plausibility.

Limitations include the lack of specific validation of the FFQ for BASE-II, a homogeneous ethnic background (European origin), and the absence of analyses to identify potential nutrient intake under-reporters (using the Goldberg method). Total nutrient intake from diet plus supplements was not assessed, preventing an evaluation of potential UL exceedances from supplement intake.

A further limitation is the lack of biochemical biomarkers of nutritional status. The present analysis assessed dietary nutrient intake based on a FFQ and therefore provides an estimate of nutrient exposure rather than a direct measure of nutritional status. An intake below the (E)AR indicates a population-level risk of inadequate intake but does not necessarily imply a clinically relevant nutrient deficiency. Conversely, an intake above the (E)AR or Reference Intake does not guarantee adequate nutritional status, as nutrient status is also influenced by absorption, metabolism, physiological requirements, and other factors. At the same time, dietary intake data remain essential for identifying population-level patterns and potential risk groups. For some nutrients, validated gold-standard biomarker methods have been developed in the past, but not for all nutrients that were reported in this study. It should be highlighted that our findings should be interpreted as indicating a risk of inadequate intake and not as confirmed clinical nutrient deficiencies. For future studies, a combination of dietary intake data and relevant biomarker data may be beneficial to assess intake data, nutritional status, and potential clinical deficiencies in detail. There are even new approaches for biomarkers of food intake (as opposed to biomarkers of nutritional status), thus possibly giving further opportunities to measure food intake more precisely [71].

The assessment of vitamin A intake is subject to limitations related to the available nutrient composition data. Although retinol equivalents (REs), retinol and β-carotene were available separately in the BLS, retinol activity equivalents values (RAEs) were not available. Therefore, direct comparison with studies or recommendations expressed in RAE should be made with caution. For comparison with the UL, the approximate sum of retinol and retinyl esters was used, as the UL applies to preformed vitamin A and not to total vitamin A from all sources.

Furthermore, it should be taken into consideration that, albeit being predominantly healthy, some participants may have a chronic disease and therefore the individual requirements for some nutrients could be higher. However, our study results apply at the population level and cannot be used for individual diagnosis; biomarker assessments would be required for clinical evaluation.

The comparison with younger BASE-II participants should be interpreted with caution, as the comparisons between age groups were primarily unadjusted and the two age groups differed in several characteristics that may influence nutrient intake, including BMI, smoking and education. These differences may have contributed to the observed differences in nutrient intake and supplement use between the groups, and residual confounding cannot be excluded. Consequently, the findings are limited to descriptive comparisons between the two BASE-II age groups. They do not allow conclusions regarding whether the observed differences are attributable to age itself, as opposed to other characteristics that differ between the groups.

It should also be taken into account that the generalisability of our findings to the wider German population is limited. BASE-II participants were independently living community-dwelling adults and may differ from the general German population in terms of health status, socioeconomic characteristics, education, lifestyle, and health-related behaviour. In particular, the study population was relatively healthy and therefore may not reflect nutrient intake patterns among older adults with multimorbidity, functional limitations, or institutionalised older adults. The quite favourable nutritional profile observed among the older BASE-II participants may also partly reflect healthy volunteer bias and may be different in an unselected older population. Thus, the findings should primarily be interpreted as describing nutrient intake and the risk of inadequate intake within this relatively healthy, independently living population rather than as estimates for the German older population as a whole.

## 5. Conclusions

Macronutrient intake in both age groups showed an overall unfavourable pattern: mean fat and protein consumption clearly exceeded the DGE reference values, while mean carbohydrate and fibre intake fell below recommended levels.

Regarding micronutrients, participants in both age groups met or substantially exceeded the DGE reference values for most nutrients. Nevertheless, a considerable proportion was at risk of inadequate intake for several nutrients—specifically calcium, magnesium, and vitamins B1 (thiamine), B2 (riboflavin), B6 (pyridoxine), B9 (folate), as well as vitamin D, with more than 15% affected in each age group. The high prevalence of insufficient vitamin D intake was expected, given that endogenous synthesis through sunlight exposure is the primary source.

Distinct age-related differences emerged: older participants showed a higher proportion at risk of inadequate calcium intake, whereas younger participants more frequently exhibited a risk for insufficient intake of vitamins B1, B2, B6, and B9. A risk of inadequate iron intake was observed exclusively among younger women. Older participants in the present study did not exhibit an overall less favourable nutrient intake than younger adults; however, it should be considered that these findings apply to a predominantly healthy, independently living population of older adults and should not be generalised to the broader older population, particularly frail or institutionalised individuals.

Yet, our findings underline the challenge of developing dietary recommendations for an increasingly heterogeneous older population. While population-level reference values provide an important basis for nutritional assessment in a healthy population, individual nutrient requirements and the ability to achieve adequate intake may vary considerably according to age, sex, body composition, physical activity, medication use, and other factors. The higher proportion of older BASE-II participants at risk of inadequate calcium intake may therefore support the need for targeted nutritional strategies rather than a uniform approach to dietary recommendations for all older adults.

Lastly, only a few studies have assessed the risk of inadequate nutrient intake using (E)ARs or probabilistic approaches. Future research should incorporate these methods more consistently to enable a more accurate evaluation of population nutrient adequacy, also with a focus on different older subpopulations. Furthermore, the relationship between dietary intake and clinical outcomes should be evaluated in older people, bearing in mind the marked heterogeneity of this population.

Since there is also a lack of studies that clearly distinguish between supplement categories according to their legal regulation within the EU, future studies should investigate this further.

## Figures and Tables

**Table 1:** Characteristics of the participants.

Characteristics		Age Group 22–36 (n = 500)	Age Group 60–84 (n = 1671)	*p*-Value
**Sex [n(%)]**	Women Men	265 (53) 235 (47)	862 (52) 809 (48)	0.58
**BMI (kg/m^2^)**	n	486	1635	
	Mean (±SD) Median (IQR) % > BMI of 25 % > BMI of 30	23 (±4) 23 (20–25) 26 6	27 (±4) 26 (24–29) 64 19	**<0.001** **<0.001** **<0.001**
**WHtR (cm/cm)**	n Mean (±SD) Median (IQR)	486 0.47 ± 0.06 0.47 (0.43–0.50)	1636 0.57 ± 0.07 0.56 (0.52–0.61)	**<0.001**
**Blood pressure ^a^** **Systolic BP (mmHG)** **Diastolic BP (mmHG)**	n Mean (± SD) Median (IQR) Mean (± SD) Median (IQR)	498 123 (±42) 121 (112–130) 80 (±43) 78 (71–84)	1669 152 (±93) 141 (130–154) 93 (±98) 83 (76–90)	**<0.001** **<0.001**
**Smoking [n(%)]**	n Current smoking No longer (for <1 year) No longer (for ≥1 year) Never smoking	499 157 (32) * 17 (3) 76 (15) 249 (50)	1660 155 (9) 15 (1) 706 (42) * 784 (47)	**<0.001**
**Alcohol consumption [n(%)]**	n Yes No	498 455 (91) 43 (9)	1666 1495 (90) 171 (10)	0.28
**Physical activity [n(%)]** ***(seldom/never physically active)***	n Yes No	488 45 (9) 443 (91)	1639 148 (9) 1491 (91)	0.90
**Education (years)**	n	410	1496	
	Mean (±SD) Median (IQR)	15 (±3) 15 (13–18)	14 (±3) 14 (12–18)	**<0.001**
**Subjective health,** **self-rated [n (%)]**	n Very good Good Fair Poor or very poor	412 104 (25) * 248 (60) 42 (10) 18 (5)	1375 182 (13) 804 (59) 314 (22) * 75 (6)	**<0.001**

BMI: Body-Mass Index; IQR: interquartile range; SD: standard deviation; WHtR: waist-to-height ratio. ^a^ Blood pressure measured seated and on the left arm. *, statistically significant difference between the groups.

**Table 2:** Percentage of younger women with a risk for an inadequate iron intake (comparison of Cut-Point Method and probability approach).

*Iron*	Average Requirement (AR)	Cut-Point Method	Probability Approach
	7 mg/day *	2%	14%
	11.2 mg/day ^#^	37%	58%

* assumed absorption rate of 18%, ^#^ assumed absorption rate of 10%.

**Table 3:** Supplement intake in the past 12 months, or for at least one month in the past 24 months.

	22–36 y			60–84 y		
	All Participants (n = 500)	Women (n = 265)	Men (n = 235)	All Participants (n = 1670)	Women (n = 861)	Men (n = 809)
Type of Supplement ^1^	Participants Taking Supplements, n (%) with 95% CI					
Overall prevalence of supplement intake (any supplement)	215 (43.0) 38.6–47.5	137 (51.7) 45.5–57.9	78 (33.2) 27.2–39.6	790 (47.3) 44.9–49.7	480 (55.7) 52.4–59.1	310 (38.3) 35.0–41.8
Food supplements (FSs) ^2^	13 (2.6) 1.4–4.4	8 (3.0) 1.3–5.9	5 (2.1) 0.7–4.9	122 (7.3) 6.1–8.7	76 (8.8) 7.0–10.9	46 (5.7) 4.2–7.5
Food supplements (FSs) ^3^	121 (24.2) 20.5–28.2	69 (26.0) 20.9–31.8	52 (22.1) 17.0–28.0	440 (26.3) 24.2–28.5	269 (31.2) 28.2–34.5	171 (21.1) 18.4–24.1
Food for special medical purposes (FSMP)	2 (0.4) 0.0–1.4	1 (0.4) 0.0–2.1	1 (0.4) 0.0–2.3	16 (1.0) 0.5–1.6	10 (1.2) 0.6–2.1	6 (0.7) 0.3–1.6
Medicinal products (MPs) with vitamins and/or minerals	19 (3.8) 2.3–5.9	15 (5.7) 3.2–9.2	4 (1.7) 0.5–4.3	93 (5.6) 4.5–6.8	68 (7.9) 6.2–9.9	25 (3.1) 2.0–4.5
MPs with herbal or other substances	11 (2.2) 1.1–3.9	9 (3.4) 1.6–6.3	2 (0.9) 0.1–3.0	84 (5.0) 4.0–6.2	47 (5.5) 4.0–7.2	37 (4.6) 3.2–6.2
Homoeopathic MPs	9 (1.8) 0.8–3.4	6 (2.3) 0.8–4.9	3 (1.3) 0.3–3.7	40 (2.4) 1.7–3.2	27 (3.1) 2.1–4.5	13 (1.6) 0.9–2.7
Multi-supplements ^4^ (of any category)	101 (20.2) 16.8–24.0	55 (20.8) 16.0–26.1	46 (19.6) 14.7–25.2	312 (18.7) 16.8–20.6	185 (21.5) 18.8–24.4	127 (15.7) 13.3–18.4
Supplements without further details	193 (38.6) 34.3–43.0	123 (46.4) 40.3–52.6	70 (29.8) 24.0–36.1	665 (39.8) 37.5–42.2	408 (47.4) 44.0–50.8	257 (31.8) 28.6–35.1

^1^ Classification according to legal regulation in the EU; ^2^ FS clearly labelled as such; ^3^ FS clearly labelled as such and supplements that are not clearly labelled as FS, but which can be assumed to be such due to their ingredients (e.g., with the algae spirulina or chlorella) and/or which are not sold as MPs; ^4^ no typical classification term; all multi-supplements were considered regardless of the classification; multi-supplements were defined as any supplement with ≥5 substances or a self-reported “multi supplement” intake.

## Data Availability

Due to concerns for participant privacy, data are available only upon reasonable request. Please contact Ludmila Müller, scientific coordinator, at lmueller@mpib-berlin.mpg.de, for additional information.

## Supplementary Materials

The supporting information can be downloaded at https://www.mdpi.com/article/10.3390/nu18183077/s1. Document S1: Further details about the assessment of adequacy or risk for inadequacy of nutrient intake; Table S1: Nutrient intake via the usual diet; Table S2: Intake of macronutrients as participants (%) with intakes >/< the respective reference value/reference value for maximum intake or Average Requirement (for protein); Table S3: Percentage of participants above the respective UL; Table S4: Percentage of the participants with vitamin and mineral intakes < AR/EAR.; Table S5: Highest prevalences of intakes <(E)AR/RV and >UL/RV in macronutrients and micronutrients.

## Abbreviations

AR	Average Requirement
BASE-II	Berlin Aging Study II
BLS	Bundeslebensmittelschlüssel, German Federal Food Code
BMI	Body-Mass Index
BVL	Bundesamt für Verbraucherschutz und Lebensmittelsicherheit, German Federal Office of Consumer Protection and Food Safety
DGE	Deutsche Gesellschaft für Ernährung, German Nutrition Society
DHA	Docosahexaenoic Acid
(E)AR	(Estimated) Average Requirement
EFSA	European Food Safety Authority
EPA	Eicosapentaenoic Acid
EPIC	European Prospective Investigation Into Cancer and Nutrition
FFQ	Food Frequency Questionnaire
EU	European Union
FSs	Food Supplements
FSMP	Food For Special Medical Purposes
GISELA	Gießener Senioren Langzeitstudie, longitudinal study in seniors from the Gießen region
IQR	Interquartile Range
IOM	Institute of Medicine
LDL	Low-Density Lipoprotein
MP	Medicinal Product
NAM	National Academy of Medicine, former Institute of Medicine
NNR	Nordic Nutrition Recommendations
NRC	National Research Council
NVS II	Nationale Verzehrsstudie II, National Nutrition Survey II
PUFAs	Polyunsaturated Fatty Acids
RDA	Recommended Dietary Allowance
RV	Reference Value
SD	Standard Deviation
SFAs	Saturated Fatty Acids
UK	United Kingdom
UL	Tolerable Upper Intake Level
WHtR	Waist-to-Height Ratio
