# Supplementary material for: Dietary Nutrient Adequacy and Supplement Use in German Community-Dwelling Older Adults Compared to a Group of Younger People: Findings from the Berlin Aging Study II (BASE-II)
Source: Nutrients. 2026 Sep 20;18(18):3077. doi: 10.3390/nu18183077 (PMC13610988; doi:10.3390/nu18183077)
Supplement: Supplementary file 1 [file nutrients-18-03077-s001.zip › Document S1.pdf]

## Document S1: Further details about the assessment of adequacy or risk for inadequacy of nutrient intake

Both the physiological requirement for nutrients and nutrient intake itself are not fixed parameters within any given population but are subject in each case to a specific distribution. In the case of requirements, a normal distribution for the sake of simplification is assumed when deriving dietary allowance RVs. These values are calculated with the aim of covering 97–98 % of the physiological requirements of the population. Based on the physiological (estimated) average requirement ((E)AR) for a nutrient, which is theoretically equal to the median of the requirement distribution, the recommended dietary allowance (RDA) for a nutrient (corresponding to the RV of the DGE) is derived by adding double the standard deviation. Since the RDA therefore represents a very high physiological requirement (97–98th requirement percentile) most persons of the population (97–98 %) consuming the nutrient on a daily basis at the level of the RDA would actually consume more of the nutrient than they require in physiological terms. For this reason, the RDA has only a limited suitability for assessing the nutrient status of a population, especially the inadequacy of nutrient intake. To identify groups at risk of having an inadequate nutrient intake, the (E)AR-Cut-Point Method by the European Food Safety Authority (EFSA) and the former IOM was therefore applied in our study [1-4]. As mentioned above, the (E)AR corresponds to the median physiological requirement for a nutrient in a population. If it is assumed that the intake of a nutrient occurs independently of the physiological need for an individual person, an intake below the (E)AR can certainly cover requirements in cases where this individual’s requirement also happens to be below the average requirement. Conversely, an intake above the (E)AR may fail to cover an individual requirement if this person happens to have a requirement that is even higher than the amounts in which the nutrient is actually consumed. Thus, an individual nutrient intake below the (E)AR is not a proof of an insufficiency for a specific person, but the proportion of a population with a nutrient intake below the (E)AR can be considered as a means of estimating the prevalence for the risk of an inadequate nutrient intake in populations [1, 3, 4]. Therefore, the lower the percentage in a population group that has a nutrient intake below the EAR, the better the nutritional status of that population group. Since the (E)AR values are derived according to gender, but not for age groups including women and men, and in many cases differ between genders, the respective lower (E)AR was chosen when analysing the risk for nutrient adequacy for the age groups.

The (E)AR Cut-Point Method should only be used in case of normally distributed nutrient requirements, hence for iron, for which it is known that the requirement distribution in menstruating women is highly skewed, this approach is not recommended. EFSA and the US National Research Council (NRC), to which the NAM belongs, recommend a probabilistic approach instead [5, 6]. The probability approach, described by the NRC in 1986, permits an estimation of the prevalence of

inadequacy within a group by comparing individual intakes to the distribution of requirements. The method applies a continuous risk-probability function to each individual's estimated intake and then averages the individual probabilities across the population or group. Like the EAR-Cut-Point-Method, the probabilistic approach does not allow a particular individual to be classified as having adequate or inadequate intakes either [3]. Only for comparison purposes with the results from the probability approach, the (E)AR Cut-Point Method was also applied to iron in younger women. Since the AR for iron depends on the absorption rate in the intestine, different ARs were used to evaluate the risk for inadequate iron intakes: the EFSA AR with an assumed high absorption rate of 18 % for premenopausal women and 16 % for men and postmenopausal women as well as a moderate absorption rate of 10 % according to [7]. The moderate absorption rate is consistent with a vegetarian diet since phytate present at relatively high levels in whole-grain cereals and legume seeds inhibits the absorption of non-haem iron [8]. This is of interest since some of the participants of BASE II reported being vegetarians. For thiamine (vitamin B1) and niacine (vitamin B3), the EARs were used despite an AR being available due to the EFSA values being related to the energy intake (mg/Megajoule) and therefore being impractical to use. For zinc, a phytate intake of 600 mg/day (medium absorption of zinc) was assumed and an AR of 7.6 and 9.3 mg/day used for females and males, respectively. Although an EAR for vitamin E exists, this EAR was not used for the analysis since the use of this cutpoint value has resulted in many reports of a high prevalence of inadequate vitamin E intakes in children in the United States, e.g. 58 % of those aged 1-2 years in one national survey and 79 % of school-age children in another study, although vitamin E deficiency is stated to be very rare and usually not caused by inadequate intakes [7]. In fact, the NAM report on the RVs for vitamin E states that ”overt symptoms of deficiency in healthy individuals consuming diets low in vitamin E have never been described” [9].


1. EFSA (European Food Safety Authority). Dietary Reference Values for nutrients: Summary report. EFSA Supporting Publications 2017, 14, e15121, 92.
2. Dietary Reference Intakes: Applications in Dietary Assessment. 4. Using the Estimated Average Requirement for Nutrient Assessment of Groups. Available online: https://www.ncbi.nlm.nih.gov/books/NBK222898/ (accessed on 5 October 2024).
3. NAM (National Academy of Medicine, former Institute of Medicine). Dietary Reference Intakes: Applications in Dietary Assessment*.* Washington DC: The National Academies Press 2000, 6.
4. Dietary Reference Intakes. The essential guide to nutrient requirements. Available online: https://www.nationalacademies.org/read/11537/chapter/1

5. EFSA (European Food Safety Authority). Scientific Opinion on principles for deriving and applying Dietary Reference Values. EFSA Journal 2010;8(3).
6. NRC. Nutrient Adequacy: Assessment Using Food Consumption Surveys. Chapter 5: The Probability Approach 1986a. Available online: https://www.ncbi.nlm.nih.gov/books/NBK217531/ (accessed on 5 October 2024 ).
7. Allen, L.H.; Carriquiry, A.L.; Murphy, S.P. Perspective: Proposed Harmonized Nutrient Reference Values for Populations. Adv Nutr 2020, 11, 469-83.
8. EFSA (European Food Safety Authority). Scientific Opinion on Dietary Reference Values for iron. EFSA Journal 2015, 13, 115.
9. Dietary Reference Intakes for Vitamin C, Vitamin E, Selenium, and Carotenoids. Available online: https://www.ncbi.nlm.nih.gov/books/NBK225483/ (accessed on 10 October 2024 ).
