# Supplementary material for: Dietary Nutrient Adequacy and Supplement Use in German Community-Dwelling Older Adults Compared to a Group of Younger People: Findings from the Berlin Aging Study II (BASE-II)
Source: Nutrients. 2026 Sep 20;18(18):3077. doi: 10.3390/nu18183077 (PMC13610988; doi:10.3390/nu18183077)
Supplement: Supplementary file 1 [file nutrients-18-03077-s001.zip › Table S1.pdf]

Table S1: Nutrient intake via the usual diet.

	Mean ±SD	P5	P10	P25	P50	P75	P95	P97.5	Min./ Max.	D-A-CH RV/RV_{MI}[a]	Reaching RV/RV_{MI} in % (Median)	p-value[b] Age	p-value[c] Gender
**Calcium (mg/d)**													
**22-36 years** *(n=490)*	947± 304	510	580	731	913	1,122	1,513	1,655	287/ 2,092	1,000	91	**< 0.01**	
**Women** *(n=260)*	857± 259	479	533	681	829	989	1,358	1,465	287/ 1,622	1,000	83		**< 0.01**
**Men** *(n=230)*	1,049± 318	563	656	836	1,012	1,248	1,663	1,748	346/ 2,092	1,000	101		
**60-84 years** *(n=1,582)*	900± 287	500	582	695	856	1,068	1,434	1,546	276/ 2,481	1,000	86		
**Women** *(n=793)*	857± 246	497	583	687	818	1,005	1,330	1,444	320/ 1,826	1,000	82		**< 0.01**
**Men** *(n=789)*	944± 318	504	580	712	900	1,134	1,495	1,660	276/ 2,481	1,000	90		
**Chloride (mg/d)**													
**22-36 y**	4,053± 1,396	2,380	2,600	3,007	3,795	4,802	6,827	7,589	1,370/ 10,860	2,300	165	> 0.05	
**Women**	3,427± 916	2,303	2,450	2,790	3,295	3,890	5,149	5,805	1,370/ 7,680	2,300	143		**< 0.01**
**Men**	4,760± 1,506	2,680	2,892	3,708	4,550	5,585	7,529	8,235	1,940/ 10,860	2,300	198		
**60-84 y**	4,028± 1,329	2,350	2,580	3,060	3,770	4,712	6,467	7,534	1,540/ 11,350	2,300	164		
**Women**	3,483± 1,005	2,194	2,410	2,770	3,310	3,995	5,386	5,929	1,540/ 8,980	2,300	144		**< 0.01**
**Men**	4,576± 1,390	2,720	3,020	3,585	4,400	5,295	7,375	8,138	1,750/ 11,350	2,300	191		
**Copper (mg/d)**													
**22-36 y**	2.6±0.7	1.5	1.8	2.1	2.5	2.9	3.8	4.0	1.0/5.8	1-1.5[d]	250	> 0.05	
**Women**	2.4±0.6	1.5	1.7	2.0	2.3	2.7	3.3	3.6	1.0/4.5	1-1.5[d]	231		**< 0.01**

	Mean ±SD	P5	P10	P25	P50	P75	P95	P97.5	Min./ Max.	D-A-CH RV/RV_{MI}[a]	Reaching RV/RV_{MI} in % (Median)	p-value[b] Age	p-value[c] Gender
**Men**	2.8±0.8	1.7	1.9	2.3	2.7	3.2	4.0	4.5	1.2/5.8	1-1.5 [d]	272		
**60-84 y**	2.5±0.7	1.5	1.7	2.0	2.5	2.9	3.9	4.3	1.1/6.4	1-1.5[d]	245		
**Women**	2.4±0.6	1.5	1.7	2.0	2.3	2.7	3.5	4.0	1.1/5.0	1-1.5[d]	231		**< 0.01**
**Men**	2.7±0.8	1.6	1.8	2.2	2.6	3.1	4.1	4.4	1.1/6.4	1-1.5[d]	259		
**Iodine (µg/d)***													
**22-36 y**	148±42	86	98	120	143	172	223	246	48/370	200	72	**< 0.01**	
**Women**	139±38	85	95	114	135	160	209	236	48/289	200	67		**< 0.01**
**Men**	158±45	87	103	127	155	179	232	258	63/370	200	78		
**60-84 y**	144±42	83	91	115	140	168	217	238	43/362	180	78		
**Women**	138±39	81	89	111	136	161	207	220	43/317	180	75		**< 0.01**
**Men**	149±45	84	95	118	146	172	230	246	47/362	180	81		
**Iron (mg/d)**													
**22-36 y**	13.9±4.1	8.5	9.2	11.1	13.3	16.0	21.3	22.8	5.6/37.1	15/10	104	**< 0.01**	
**Women**	12.3±2.8	7.9	8.8	10.3	12.1	13.9	17.4	18.0	5.6/22.9	15	81		**< 0.01**
**Men**	15.8±4.6	9.2	10.4	12.8	15.1	18.3	22.8	24.4	6.4/37.1	10	151		
**60-84 y**	14.2±4.2	8.8	9.6	11.3	13.6	16.4	21.9	24.6	4.8/36.9	10	136		
**Women**	12.8±3.5	8.4	9.0	10.5	12.3	14.5	19.1	21.8	4.8/29.7	10	123		**< 0.01**
**Men**	15.6±4.4	9.6	10.5	12.4	15.2	18.0	23.4	25.7	4.8/36.9	10	151		
**Magnesium (mg/d)**													
**22-36 y**	351±95	215	240	283	338	411	520	570	147/819	300/350	107	> 0.05	
**Women**	313±71	206	224	266	306	355	439	457	147/583	300	102		**< 0.01**
**Men**	395±101	238	270	324	392	450	569	597	170/819	350	112		
**60-84 y**	356±99	222	244	285	344	408	539	593	136/787	300/350	106		
**Women**	320±79	211	232	267	309	359	466	507	136/724	300	103		**< 0.01**
**Men**	393±103	242	269	321	382	452	576	627	165/787	350	109		
**Manganese (mg/d)**													
**22-36 y**	5.1±2.3	2.7	2.9	3.6	4.5	6.1	9.7	11.2	1.7/18.8	2-5[e]	227	> 0.05	
**Women**	5.0±2.4	2.5	2.9	3.5	4.3	5.8	9.7	11.5	1.7/18.8	2-5[e]	217		> 0.05
**Men**	5.3±2.2	2.8	3.0	3.6	4.9	6.4	9.9	11.0	1.7/13.4	2-5[e]	244		
**60-84 y**	5.4±2.5	2.7	3.0	3.7	4.8	6.2	10.6	12.2	1.2/23.9	2-5[e]	240		

	Mean ±SD	P5	P10	P25	P50	P75	P95	P97.5	Min./ Max.	D-A-CH RV/RV_{MI}[a]	Reaching RV/RV_{MI} in % (Median)	p-value[b] Age	p-value[c] Gender
**Women**	4.9±2.2	2.5	2.8	3.4	4.4	5.7	9.4	11.2	1.2/19.4	2-5[e]	217		**< 0.01**
**Men**	5.9±2.7	2.8	3.2	4.1	5.3	6.9	11.4	12.7	1.5/23.9	2-5[e]	267		
**Phosphorus (mg/d)**													
**22-36 y**	1,336± 395	759	873	1,045	1,302	1,578	2,039	2,185	441/ 3,036	700	186	> 0.05	
**Women**	1,148± 286	714	795	967	1,130	1,322	1,671	1,767	441/ 2,035	700	161		**< 0.01**
**Men**	1,548± 395	939	1,067	1,314	1,526	1,779	2,196	2,362	619/ 3,036	700	218		
**60-84 y**	1,357± 395	828	917	1,067	1,303	1,574	2,095	2,284	460/ 3,882	700	186		
**Women**	1,203± 311	753	846	1,000	1,165	1,371	1,785	1,953	460/ 2,510	700	166		**< 0.01**
**Men**	1,513± 410	928	1,017	1,222	1,477	1,736	2,234	2,434	504/ 3,882	700	211		
**Potassium (mg/d)**													
**22-36 y**	3,013± 851	1,820	1,978	2,408	2,901	3,510	4,557	5,023	1,014/ 7,050	4,000	73	**< 0.01**	
**Women**	2,724± 680	1,702	1,906	2,270	2,664	3,184	3,890	4,012	1,014/ 5,737	4,000	67		**< 0.01**
**Men**	3,339± 907	1,918	2,251	2,691	3,269	3,862	5,006	5,210	1,330/ 7,050	4,000	82		
**60-84 y**	3,296± 919	2,069	2,258	2,662	3,181	3,755	4,977	5,591	1,158/ 9,275	4,000	80		
**Women**	3,041± 780	1,979	2,159	2,523	2,939	3,452	4,382	5,003	1,158/ 7,635	4,000	73		**< 0.01**
**Men**	3,552± 975	2,198	2,404	2,933	3,442	4,038	5,303	5,812	1,457/ 9,275	4,000	86		
**Sodium (mg/d)**													

	Mean ±SD	P5	P10	P25	P50	P75	P95	P97.5	Min./ Max.	D-A-CH RV/RV_{MI}[a]	Reaching RV/RV_{MI} in % (Median)	p-value[b] Age	p-value[c] Gender
**22-36 y**	2,384± 900	1,314	1,447	1,717	2,214	2,849	4,118	4,632	694/ 6,454	1,500	148	> 0.05	
**Women**	1,944± 568	1,265	1,377	1,542	1,845	2,235	3,036	3,496	694/ 4,663	1,500	123		**< 0.01**
**Men**	2,882± 947	1,588	1,744	2,241	2,754	3,461	4,606	5,009	1085/ 6,454	1,500	184		
**60-84 y**	2,394± 847	1,341	1,498	1,783	2,236	2,834	3,963	4,655	835/ 7,394	1,500	149		
**Women**	2,024± 622	1,243	1,368	1,605	1,912	2,325	3,171	3,577	835/ 5,607	1,500	127		**< 0.01**
**Men**	2,767± 880	1,617	1,807	2,168	2,639	3,212	4,555	5,062	933/ 7,394	1,500	176		
Zinc (mg/d)													
**22-36 y**	12.0±3.6	7.2	8.0	9.3	11.5	14.1	18.4	19.7	3.4/29.4	7-10/11-16[f]	134	**< 0.01**	
**Women**	10.2±2.4	6.6	7.6	8.6	9.9	11.7	14.4	15.9	3.4/18.2	7-10[f]	141		**< 0.01**
**Men**	14.1±3.7	8.5	9.9	11.7	13.7	16.5	19.9	21.2	5.4/29.4	11-16[f]	125		
**60-84 y**	11.7±3.6	7.1	7.9	9.1	11.0	13.5	18.0	20.6	4.2/36.7	7-10/11-16[f]	128		
**Women**	10.3±2.7	6.6	7.3	8.5	9.8	11.7	15.4	17.1	4.6/22.9	7-10[f]	140		**< 0.01**
**Men**	13.1±3.8	7.9	8.7	10.3	12.8	15.0	19.9	22.2	4.2/36.7	11-16[f]	116		
Vitamin A (Retinolequivalents/RE) (mg/d)													
**22-36 y**	1.6±0.7	0.8	0.9	1.1	1.4	1.9	2.7	3.0	0.4/7.1	0.8/1	160	**< 0.01**	
**Women**	1.4±0.5	0.8	0.9	1.0	1.3	1.6	2.4	2.5	0.4/3.0	0.8	161		> 0.05
**Men**	1.8±0.8	0.9	1.0	1.3	1.6	2.1	3.2	4.0	0.6/7.1	1	155		
**60-84 y**	1.9±0.8	0.9	1.0	1.3	1.7	2.2	3.3	3.8	0.5/13.6	0.8/1	193		
**Women**	1.7±0.7	0.9	1.0	1.3	1.6	2.0	3.1	3.5	0.5/5.2	0.8	202		**< 0.01**
**Men**	2.0±0.9	0.9	1.1	1.4	1.8	2.3	3.5	4.1	0.5/13.6	1	182		
Vitamin A (mg/d) (calculated approx. Retinol+Retinylesters: RE minus β-Carotene, for comparison with the Tolerable Upper Intake Level/UL)													
**22-36 y**	0.9±0.6	0.4	0.5	0.6	0.8	1.1	1.9	2.2	0.1/6.6	--	--	**< 0.01**	
**Women**	0.8±0.4	0.4	0.5	0.5	0.7	1.0	1.5	1.7	0.1/2.2	--	--		**< 0.01**

	Mean ±SD	P5	P10	P25	P50	P75	P95	P97.5	Min./ Max.	D-A-CH RV/RV_{MI}[a]	Reaching RV/RV_{MI} in % (Median)	p-value[b] Age	p-value[c] Gender
**Men**	1.1±0.7	0.5	0.6	0.7	0.9	1.3	2.3	3.1	0.3/6.6	--	--		
**60-84 y**	1.2±0.7	0.4	0.5	0.7	1.1	1.5	2.4	2.9	0.2/12.2	--	--		
**Women**	1.1±0.6	0.4	0.5	0.7	1.0	1.3	2.2	2.6	0.2/4.5	--	--		**< 0.01**
**Men**	1.3±0.8	0.5	0.6	0.8	1.2	1.6	2.6	3.1	0.2/12.2	--	--		
**Vitamin A (Retinol) (mg/d)**													
**22-36 y**	0.8±0.5	0.3	0.4	0.5	0.7	0.9	1.8	2.1	0.1/6.6	--	--	**< 0.01**	
**Women**	0.7±0.3	0.3	0.4	0.4	0.6	0.8	1.4	1.6	0.1/2.2	--	--		**< 0.01**
**Men**	1.0±0.7	0.4	0.5	0.6	0.8	1.1	2.1	2.9	0.2/6.6	--	--		
**60-84 y**	1.1±0.7	0.4	0.4	0.6	0.9	1.3	2.3	2.8	0.1/12.1	--	--		
**Women**	0.9±0.6	0.3	0.4	0.6	0.9	1.1	2.0	2.5	0.1/4.4	--	--		**< 0.01**
**Men**	1.2±0.8	0.4	0.5	0.7	1.1	1.5	2.5	3.0	0.2/12.1	--	--		
**Vitamin A (β-Carotene) (mg/d)**													
**22-36 y**	3.8±1.8	1.8	2.0	2.5	3.3	4.6	7.4	8.3	1.0/14.2	--	--	> 0.05	
**Women**	3.6±1.6	1.6	1.9	2.5	3.2	4.3	6.8	7.6	1.0/10.9	--	--		> 0.05
**Men**	3.9±2.0	1.9	2.2	2.7	3.5	4.6	7.8	9.8	1.2/14.2	--	--		
**60-84 y**	3.9±2.0	1.8	2.1	2.6	3.4	4.6	7.8	9.0	0.8/21.4	--	--		
**Women**	3.9±1.9	1.8	2.1	2.6	3.5	4.6	7.8	9.0	1.1/15.7	--	--		> 0.05
**Men**	3.9±2.1	1.8	2.1	2.6	3.4	4.6	7.9	9.0	0.8/21.4	--	--		
**Vitamin B1 (Thiamine) (mg/d)**													
**22-36 y**	1.3±0.5	0.7	0.7	0.9	1.1	1.5	2.2	2.5	0.4/3.7	1/1.2[g]	107	**< 0.01**	
**Women**	1.0±0.3	0.6	0.7	0.8	1.0	1.1	1.5	1.6	0.4/1.9	1	95		**< 0.01**
**Men**	1.5±0.5	0.8	0.9	1.2	1.5	1.8	2.5	2.8	0.6/3.7	1.2[g]	122		
**60-84 y**	1.3±0.5	0.7	0.8	0.9	1.2	1.5	2.1	2.5	0.4/4.9	1/1.1[g]	113		
**Women**	1.1±0.3	0.6	0.7	0.8	1.0	1.2	1.6	1.9	0.4/3.6	1	101		**< 0.01**
**Men**	1.5±0.5	0.8	0.9	1.1	1.4	1.8	2.4	2.8	0.6/4.9	1.1[g]	129		
**Vitamin B12 (Cobalamin) (µg/d)**													
**22-36 y**	5.6±2.7	2.3	2.6	3.6	5.0	7.4	10.8	12.0	0.6/19.3	4	125	**< 0.01**	
**Women**	4.2±1.6	2.0	2.5	3.1	4.0	5.2	7.4	7.9	0.6/9.2	4	101		**< 0.01**
**Men**	7.2±2.9	3.1	3.7	5.1	7.1	9.1	12.0	14.2	1.4/19.3	4	177		
**60-84 y**	6.7±2.9	3.1	3.7	4.8	6.2	8.2	11.8	13.3	0.9/36.0	4	155		

	Mean ±SD	P5	P10	P25	P50	P75	P95	P97.5	Min./ Max.	D-A-CH RV/RV_{MI}[a]	Reaching RV/RV_{MI} in % (Median)	p-value[b] Age	p-value[c] Gender
**Women**	5.5±2.0	2.8	3.3	4.2	5.3	6.5	9.0	10.6	1.0/15.5	4	132		**< 0.01**
**Men**	8.0±3.2	3.9	4.7	6.0	7.6	9.6	13.1	14.9	0.9/36.0	4	189		
**Vitamin B2 (Riboflavine) (mg/d)**													
**22-36 y**	1.5±0.5	0.8	1.0	1.2	1.5	1.8	2.5	2.6	0.6/3.6	1.1/1.4	123	**< 0.01**	
**Women**	1.3±0.4	0.8	0.9	1.1	1.3	1.5	2.0	2.1	0.6/2.4	1.1	120		> 0.05
**Men**	1.8±0.5	1.0	1.2	1.4	1.8	2.1	2.7	2.9	0.6/3.6	1.4	127		
**60-84 y**	1.7±0.5	1.0	1.1	1.3	1.6	1.9	2.5	2.9	0.6/5.8	1.0/1.3	140		
**Women**	1.5±0.4	0.9	1.0	1.2	1.4	1.7	2.2	2.4	0.6/3.1	1.0	143		**< 0.01**
**Men**	1.8±0.6	1.1	1.2	1.4	1.8	2.1	2.8	3.1	0.6/5.8	1.3	136		
**Vitamin B3 (Niacinequivalents/NE) (mg/d)**													
**22-36 y**	29.2±10.4	16.0	18.2	21.6	27.3	35.7	48.6	53.6	9.8/76.3	12/15[g]	208	**< 0.01**	
**Women**	23.6±6.2	14.1	16.3	19.4	22.6	27.3	35.3	37.3	9.8/46.4	12[g]	188		**< 0.01**
**Men**	35.5±10.6	19.9	22.9	27.9	34.9	42.0	53.9	57.5	12.4/76.3	15[g]	233		
**60-84 y**	30.9±10.1	18.0	20.0	24.0	29.3	36.1	50.1	55.3	10.2/97.4	11/14[g]	237		
**Women**	26.3±7.0	16.6	18.4	21.6	25.5	30.0	38.5	44.2	10.2/61.2	11[g]	232		**< 0.01**
**Men**	35.6±10.5	20.9	23.9	28.5	34.1	41.2	53.9	58.6	10.9/97.4	14[g]	243		
**Vitamin B5 (Pantothenic acid) (mg/d)**													
**22-36 y**	4.9±1.4	2.9	3.3	3.9	4.8	5.9	7.4	8.0	1.9/11.5	5	95	**< 0.01**	
**Women**	4.3±1.0	2.7	3.0	3.6	4.2	5.0	6.3	6.6	1.9/7.4	5	84		**< 0.01**
**Men**	5.6±1.4	3.4	3.9	4.6	5.5	6.5	8.0	8.5	2.1/11.5	5	111		
**60-84 y**	5.2±1.5	3.2	3.6	4.2	5.0	6.1	8.0	8.7	1.7/14.6	5	101		
**Women**	4.7±1.2	3.0	3.4	3.9	4.5	5.4	6.9	7.6	1.7/10.3	5	91		**< 0.01**
**Men**	5.8±1.6	3.5	4.0	4.7	5.6	6.6	8.6	9.7	2.3/14.6	5	112		
**Vitamin B6 (Pyridoxine) (mg/d)**													
**22-36 y**	1.6±0.5	0.9	1.0	1.2	1.5	1.9	2.6	2.9	0.6/4.3	1.4/1.6	104	**< 0.01**	
**Women**	1.4±0.3	0.8	1.0	1.1	1.3	1.6	2.0	2.1	0.6/2.4	1.4	94		**< 0.01**
**Men**	1.9±0.6	1.1	1.3	1.6	1.9	2.3	2.9	3.0	0.8/4.3	1.6	117		
**60-84 y**	1.7±0.5	1.0	1.1	1.4	1.6	2.0	2.7	3.1	0.7/4.6	1.4/1.6	110		
**Women**	1.5±0.4	0.96	1.0	1.3	1.5	1.7	2.3	2.6	0.7/3.5	1.4	104		**< 0.01**
**Men**	2.0±0.6	1.2	1.3	1.6	1.9	2.3	3.0	3.3	0.7/4.6	1.6	119		

	Mean ±SD	P5	P10	P25	P50	P75	P95	P97.5	Min./ Max.	D-A-CH RV/RV_{MI}[a]	Reaching RV/RV_{MI} in % (Median)	p-value[b] Age	p-value[c] Gender
**Vitamin B7 (Biotin) (µg/d)**													
**22-36 y**	47.2±13.9	26.6	30.7	37.6	45.5	55.7	72.6	77.6	17.9/ 110.8	40	114	**< 0.01**	
**Women**	41.9±10.7	24.7	27.8	34.4	40.6	49.3	61.7	64.7	17.9/ 69.9	40	102		**< 0.01**
**Men**	53.1±14.7	30.5	36.5	42.7	52.6	62.1	77.7	82.1	17.9/ 110.8	40	132		
**60-84 y**	49.9±14.9	29.5	33.5	39.4	47.4	58.1	76.7	85.7	17.1/ 151.0	40	119		
**Women**	45.5±12.3	28.1	31.2	37.3	44.0	52.4	68.9	74.9	17.1/ 106.4	40	110		**< 0.01**
**Men**	54.3±16.0	32.7	36.4	43.3	52.5	62.4	82.4	92.9	20.5/ 151	40	131		
**Vitamin B9 (Folateequivalents/FE) (µg/d)**													
**22-36 y**	235±67	137	155	188	225	274	355	377	75/547	300	75	**< 0.01**	
**Women**	220±60	133	147	179	212	255	337	354	75/398	300	71		**< 0.01**
**Men**	252±72	145	167	203	242	290	367	402	103/547	300	81		
**60-84 y**	253±75	153	171	203	242	290	385	436	91/824	300	81		
**Women**	236±67	148	164	196	227	268	347	388	91/734	300	76		**< 0.01**
**Men**	270±80	164	183	219	258	308	415	463	11/824	300	86		
**Vitamin C (mg/d)**													
**22-36 y**	138±61	67	76	97	127	166	245	283	47/581	95/110	125	**< 0.01**	
**Women**	133±59	63	73	89	124	164	233	261	47/582	95	130		> 0.05
**Men**	145±63	73	83	102	132	169	265	332	48/424	110	120		
**60-84 y**	149±70	75	86	107	135	171	265	323	44/755	95/110	131		
**Women**	147±67	74	85	106	133	172	258	301	44/720	95	140		**< 0.01**
**Men**	151±73	75	86	109	137	171	270	345	45/755	110	124		
**Vitamin D (µg/day)**													
**22-36 y**	4.1±2.7	1.5	1.7	2.4	3.5	4.9	9.9	10.9	0.3/19.4	20	17	**< 0.01**	
**Women**	3.3±1.8	1.3	1.5	2.1	2.9	4.0	6.6	7.7	0.3/12.4	20	14		**< 0.01**

	Mean ±SD	P5	P10	P25	P50	P75	P95	P97.5	Min./ Max.	D-A-CH RV/RV_{MI}[a]	Reaching RV/RV_{MI} in % (Median)	p-value[b] Age	p-value[c] Gender
**Men**	5.1±3.1	1.9	2.3	3.0	4.2	6.2	10.9	13.3	1.0/19.4	20	21		
**60-84 y**	5.1±2.9	1.8	2.2	3.1	4.4	6.2	10.5	12.3	0.6/19.3	20	22		
**Women**	4.0±1.9	1.6	1.9	2.8	3.6	5.1	7.3	8.8	0.6/13.7	20	18		**< 0.01**
**Men**	6.2±3.3	2.2	2.6	4.0	5.2	8.7	12.0	15.8	1.2/19.3	20	26		
**Vitamin E (Tocopherolequivalents/TE) (mg/d)**													
**22-36 y**	14.1±4.4	8.3	9.1	11.1	13.5	16.1	21.8	24.0	5.8/39.0	12/14	105	**< 0.01**	
**Women**	12.5±3.2	8.0	8.6	10.2	12.3	14.1	18.8	20.0	5.8/23.1	12	103		**0.01**
**Men**	15.9±4.8	9.9	10.8	12.5	15.4	18.4	24.2	26.4	7.4/39.0	14[g]	110		
**60-84 y**	14.4±4.7	8.6	9.6	11.3	13.6	16.6	23.0	26.2	5.0/45.6	11/12	118		
**Women**	13.2±4.2	8.2	8.9	10.5	12.4	15.1	20.9	23.1	5.0/44.4	11[g]	113		**< 0.01**
**Men**	15.7±4.9	9.6	10.4	12.5	14.9	18.1	24.9	28.3	5.5/45.6	12[g]	124		
**Vitamin E (α-Tocopherol) (mg/d)**													
**22-36 y**	11.4±3.3	7.1	7.8	9.1	11.0	13.1	17.5	19.3	5.3/29.2	--	--	**< 0.01**	
**Women**	10.4±2.6	6.4	7.2	8.6	10.2	12.0	15.0	15.7	5.3/18.5	--	--		**< 0.01**
**Men**	12.6±3.7	8.3	8.9	10.0	12.0	14.1	19.6	22.3	6.3/29.2	--	--		
**60-84 y**	12.0±3.8	7.3	8.1	9.5	11.3	13.6	19.0	21.6	4.5/40.2	--	--		
**Women**	11.3±3.5	7.2	7.7	9.1	10.7	12.7	17.4	20.2	4.5/40.0	--	--		**< 0.01**
**Men**	12.7±3.9	7.9	8.6	10.0	12.1	14.2	20.1	22.9	4.9/40.2	--	--		
**Vitamin K (Phylloquinone) (µg/d)**													
**22-36 y**	314±92	187	214	250	304	363	488	515	141/ 795	60/70	462	**< 0.01**	
**Women**	282±74	175	200	228	271	322	431	485	141/ 564	60	452		> 0.05
**Men**	350±97	216	237	287	338	405	512	556	143/ 795	70	483		
**60-84 y**	330±107	205	222	261	311	375	522	591	122/ 1,131	65/80	433		
**Women**	305±94	197	212	244	289	341	476	536	122/ 1,001	65	444		**< 0.01**
**Men**	356±112	222	240	286	337	401	556	636	134/	80	421		

	Mean ±SD	P5	P10	P25	P50	P75	P95	P97.5	Min./ Max.	D-A-CH RV/RV_{MI}[a]	Reaching RV/RV_{MI} in % (Median)	p-value[b] Age	p-value[c] Gender
									1,131				
**Energy (kcal/d)**													
**22-36 y**	2,319± 754	1,323	1,531	1,776	2,181	2,733	3,664	3,954	908/ 6,184	2,100/ 2,700[h]	93	**< 0.01**	
**Women**	1,968± 493	1,217	1,376	1,639	1,899	2,239	2,875	3,008	908/ 3,892	2,100[h]	90		**< 0.05**
**Men**	2,717± 801	1,576	1,815	2,172	2,627	3,166	3,972	4,472	1,100/ 6,184	2,700[h]	97		
**60-84 y**	2,253± 706	1,316	1,483	1,745	2,143	2,640	3,598	3,979	872/ 5,896	1,900/ 2,500[h]	98		
**Women**	1,960± 561	1,254	1,379	1,573	1,859	2,224	2,979	3,349	872/ 5,123	1,900[h]	98		> 0.05
**Men**	2,548± 714	1,581	1,702	2,043	2,468	2,948	3,865	4,205	880/ 5,896	2,500[h]	99		
**Protein (g/d)**													
**22-36 y**	81.1± 31.5	42.1	48.0	58.6	75.0	97.0	142.1	155.6	25.0/ 249.9	48/57	145	**< 0.01**	
**Women**	64.3± 17.7	37.4	44.8	52.5	61.7	75.1	94.5	101.6	25.0/ 132.2	48	129		**< 0.01**
**Men**	100.0± 32.9	55.9	61.0	77.1	94.8	119.2	156.9	174.9	31.8/ 249.9	57	166		
**60-84 y**	80.0± 27.3	45.9	51.2	61.1	75.2	93.7	130.2	144.0	23.7/ 277.8	57/67[g]	122		
**Women**	68.1± 19.8	41.7	47.0	54.9	65.7	76.8	102.4	117.2	23.7/ 196.4	57[g]	115		**< 0.01**
**Men**	91.9± 28.6	54.9	60.2	72.4	89.0	106.1	139.3	158.6	23.7/ 277.8	67[g]	133		
**Carbohydrates (g/d)**													
**22-36 y**	245±91	139	154	184	224	287	438	485	98/786	--	--	--	--
**Women**	217±63	129	148	173	207	253	329	371	98/467	--	--	--	--
**Men**	276±107	148	163	206	252	318	487	565	105/786	--	--	--	--

	Mean ±SD	P5	P10	P25	P50	P75	P95	P97.5	Min./ Max.	D-A-CH RV/RV_{MI}[a]	Reaching RV/RV_{MI} in % (Median)	p-value[b] Age	p-value[c] Gender
**60-84 y**	225±78	128	142	169	212	264	372	417	72/790	--	--	--	--
**Women**	205±70	124	135	157	191	234	325	389	72/651	--	--	--	--
**Men**	246±81	140	155	190	234	290	397	440	82/790	--	--	--	--
**Carbohydrates (En%)**													
**22-36 y**	42±6	32	35	38	42	46	53	54	23/66	(>)50 En%	85	**< 0.01**	
**Women**	44±6	36	38	40	44	47	53	56	27/66	(>)50 En%	88		**< 0.01**
**Men**	40±6	30	33	37	39	44	51	54	23/57	(>)50 En%	78		
**60-84 y**	40±6	31	33	36	40	44	50	52	17/62	(>)50 En%	80		
**Women**	42±6	32	35	38	42	45	51	53	25/62	(>)50 En%	83		**< 0.01**
**Men**	38±6	29	31	35	38	42	49	50	17/59	(>)50 En%	77		
**Dietary fibre (g/d)**													
**22-36 y**	22.2±7.1	12.8	14.4	16.8	21.3	25.9	35.9	38.9	8.9/60.9	(≥)30	71	**< 0.05**	
**Women**	20.9±5.8	12.5	14.0	16.3	20.7	24.1	31.7	35.0	8.9/41.8	(≥)30	69		**< 0.01**
**Men**	23.7±8.2	13.4	14.8	17.0	22.6	28.1	38.9	41.1	10.4/60.9	(≥)30	75		
**60-84 y**	23.2±7.7	13.3	14.9	17.9	21.9	27.0	37.0	42.0	6.8/66.7	(≥)30	73		
**Women**	22.1±7.1	13.0	14.4	17.3	21.0	25.5	35.2	40.1	6.8/60.0	(≥)30	70		**< 0.01**
**Men**	24.3±8.1	13.7	15.5	18.6	23.2	28.7	38.9	43.4	8.2/66.7	(≥)30	77		
**Fat (g/d)**													
**22-36 y**	101.4± 34.2	54.3	63.2	76.8	96.8	121.3	164.3	175.4	29.5/ 285.7	--	--	--	--
**Women**	84.2± 23.7	50.9	57.3	68.1	81.1	96.8	126.8	135.5	29.5/ 194.4	--	--	--	--
**Men**	120.9± 33.9	68.2	81.1	99.3	116.2	145.0	174.7	184.3	50.6/ 285.7	--	--	--	--
**60-84 y**	100.8± 34.6	56.9	62.9	76.7	94.9	117.8	165.0	188.3	33.4/ 306.7	--	--	--	--
**Women**	86.5± 26.9	52.7	57.9	68.5	81.9	98.9	135.3	149.8	33.4/ 277.8	--	--	--	--
**Men**	115.1±	69.0	75.3	91.3	110.5	132.7	184.7	206.8	39.4/	--	--	--	--

	Mean ±SD	P5	P10	P25	P50	P75	P95	P97.5	Min./ Max.	D-A-CH RV/RV_{MI}[a]	Reaching RV/RV_{MI} in % (Median)	p-value[b] Age	p-value[c] Gender
	35.6								306.7				
**Fat (En%)**													
**22-36 y**	41±6	31	34	38	41	44	50	51	16/55	(<)30 En%	137	**< 0.05**	
**Women**	40±5	31	34	37	40	43	49	50	16/55	(<)30 En%	134		**< 0.01**
**Men**	42±6	31	34	38	43	46	51	52	23/55	(<)30 En%	142		
**60-84 y**	42±5	33	35	39	42	45	50	51	18/62	(<)30 En%	139		
**Women**	41±5	33	35	38	41	44	49	50	18/58	(<)30 En%	137		**< 0.01**
**Men**	42±5	33	35	39	43	46	50	51	23/62	(<)30 En%	142		
**Saturated fatty acids (g/d)**													
**22-36 y**	42.2±15.1	21.6	25.0	31.8	39.1	50.8	70.8	76.2	8.6/ 121.4	--	--	--	--
**Women**	35.6±11.3	19.6	22.9	28.4	33.9	41.0	57.4	61.4	8.6/ 82.7	--	--	--	--
**Men**	49.7±15.4	26.5	32.9	39.1	47.7	59.4	75.2	83.8	18.1/ 121.4	--	--	--	--
**60-84 y**	41.2±15.2	22.1	24.7	30.7	38.2	48.8	69.9	80.8	11.8/ 126.3	--	--	--	--
**Women**	35.7±12.1	20.5	22.7	27.6	33.6	41.4	59.3	66.2	11.8/ 110.1	--	--	--	--
**Men**	46.7±15.9	25.6	29.1	36.0	43.9	54.8	77.0	87.7	15.4/ 126.3	--	--	--	--
**Saturated fatty acids (En%) (RV_{MI})**													
**22-36 y**	16.4±2.9	11.6	12.9	14.6	16.5	18.4	20.9	21.6	5.8/24.9	<7-10 En%	235-165	> 0.05	
**Women**	16.2±2.8	11.9	13.1	14.6	16.2	18.2	20.8	21.4	5.8/23.2	<7-10 En%	231-162		> 0.05
**Men**	16.7±2.9	11.6	12.6	14.7	16.8	18.7	21.2	21.7	8.9/24.9	<7-10 En%	239-168		
**60-84 y**	16.4±2.6	11.9	13.1	14.7	16.5	18.2	20.6	21.4	7.2/25.6	<7-10 En%	235-165		
**Women**	16.4±2.5	12.0	13.2	14.7	16.4	17.9	20.6	21.5	7.2/24.2	<7-10 En%	235-164		> 0.05
**Men**	16.5±2.6	11.9	12.9	14.7	16.5	18.4	20.6	21.4	9.1/25.6	<7-10 En%	236-165		
**Polyunsaturated fatty acids (g/d)**													
**22-36 y**	16.9±6.6	8.9	10.0	12.4	15.7	19.8	29.8	32.6	6.0/60.5	--	--	--	--

	Mean ±SD	P5	P10	P25	P50	P75	P95	P97.5	Min./ Max.	D-A-CH RV/RV_{MI}[a]	Reaching RV/RV_{MI} in % (Median)	p-value[b] Age	p-value[c] Gender
**Women**	13.9±4.3	8.4	9.1	11.0	13.5	16.1	22.4	25.0	6.0/31.2	--	--	--	--
**Men**	20.3±7.1	11.0	13.1	15.6	18.7	23.5	32.8	36.4	7.6/60.5	--	--	--	--
**60-84 y**	17.1±6.5	9.4	10.5	12.7	15.9	19.6	30.0	34.3	5.6/52.7	--	--	--	--
**Women**	14.7±5.1	8.5	9.6	11.3	13.7	16.9	23.7	27.6	5.6/48.4	--	--	--	--
**Men**	19.6±6.9	11.1	12.4	14.9	18.2	22.9	33.0	36.6	7.1/52.7	--	--	--	--
**Polyunsaturated fatty acids (En%) (RV_{MI})**													
**22-36 y**	6.6±1.6	4.4	5.0	5.6	6.4	7.5	9.5	10.3	2.9/14.9	up to 10 En%	64	**< 0.01**	
**Women**	6.4±1.4	4.3	4.9	5.5	6.3	7.1	9.0	10.0	3.2/11.7	up to 10 En%	63		**< 0.05**
**Men**	6.8±1.7	4.5	5.0	5.6	6.6	7.9	9.6	10.5	2.9/14.9	up to 10 En%	66		
**60-84 y**	6.9±1.6	4.8	5.2	5.8	6.7	7.7	9.8	10.7	2.4/14.4	up to 10 En%	67		
**Women**	6.8±1.5	4.9	5.2	5.7	6.6	7.6	9.6	10.2	2.4/14.4	up to 10 En%	66		> 0.05
**Men**	7.0±1.7	4.7	5.0	5.8	6.8	7.9	10.1	11.1	3.1/14.1	up to 10 En%	68		
**Eicosapentaenoic+Docosahexaenoic acid/EPA+DHA (mg/d) (Reference value by European Food Safety Authority EFSA)**													
**22-36 y**	311±259	71	87	147	237	395	907	979	28/ 1,872	250[i]	95	**< 0.01**	
**Women**	232±171	60	76	113	188	286	548	650	28/ 1,116	250[i]	75		**< 0.01**
**Men**	400±309	88	128	186	326	470	960	1,154	41/ 1,872	250[i]	130		
**60-84 y**	424±286	109	143	231	357	515	943	1,070	29/ 1,769	250[i]	143		
**Women**	326±183	95	116	209	279	445	620	807	29/ 1,237	250[i]	111		**< 0.01**
**Men**	523±332	136	166	308	420	793	1,025	1,484	38/ 1,769	250[i]	168		

	Mean ±SD	P5	P10	P25	P50	P75	P95	P97.5	Min./ Max.	D-A-CH RV/RV$_{MI}$[a]	Reaching RV/RV$_{MI}$ in % (Median)	p-value[b] Age	p-value[c] Gender
**Salt (g/d) (RV$_{MI}$)**													
**22-36 y**	5.7±2.1	3.2	3.5	4.1	5.3	6.9	10.0	10.8	1.7/15.5	(<)6	88	> 0.05	
**Women**	4.7±1.4	3.1	3.3	3.7	4.4	5.4	7.3	8.3	1.7/11.6	(<)6	74		**< 0.01**
**Men**	6.9±2.3	3.8	4.1	5.3	6.6	8.2	10.8	11.8	2.6/15.5	(<)6	110		
**60-84 y**	5.7±2.0	3.2	3.6	4.3	5.3	6.8	9.4	11.0	2.0/17.2	(<)6	89		
**Women**	4.8±1.5	2.9	3.3	3.8	4.5	5.5	7.7	8.5	2.0/13.2	(<)6	75		**< 0.01**
**Men**	6.6±2.1	3.8	4.3	5.1	6.3	7.7	10.9	12.0	2.3/17.2	(<)6	105		
**Alcohol (g/d) (RV$_{MI}$)**													
**22-36 y**	11.1±12.3	0.7	1.0	2.3	7.1	15.2	40.2	48.6	0.5/81.0	(<)10/(<)20	47	**< 0.01**	
**Women**	8.9±10.5	0.6	0.8	1.9	5.3	12.6	28.7	46.1	0.5/62.9	(<)10	53		**< 0.01**
**Men**	13.6±13.7	1.0	1.3	3.9	8.6	18.8	42.7	49.3	0.5/81.0	(<)20	43		
**60-84 y**	15.0±18.4	0.8	1.2	2.9	8.5	20.0	52.8	66.8	0.4/166.6	(<)10/(<)20	57		
**Women**	10.0±12.5	0.7	1.0	2.2	5.4	12.8	35.4	48.4	0.4/93.4	(<)10	54		**< 0.01**
**Men**	20.0±21.8	0.9	1.6	4.9	12.7	26.3	61.9	77.8	0.4/166.6	(<)20	64		

RV: Reference value, RV$_{MI}$: Reference value for maximum intake, -- No reference value exists, En%: energy %

[a]D-A-CH reference values: joint reference values for the German-speaking European regions (Germany, Austria, Switzerland)

[b]the p-value refers to the difference in reaching the reference values in % where there was a reference value available. In case of a reference value not being available, the absolute intake values were compared. The difference between older vs. younger participants was tested using the Mann-Whitney-U test.

[c]the p-value refers to the difference in reaching the reference values in % where there was a reference value available. In case of a reference value not being available, the absolute intake values were compared. The difference between women and men in the respective age groups was tested using the Kruskal-Wallis test with subsequent Bonferroni correction.

[d]1 mg was used to calculate the attainment of the reference value

[e]2 mg was used to calculate the attainment of the reference value

[f]a mixed diet with a low phytate intake was assumed (reference values 7 and 11 mg)

[g]since only a small proportion of the participants were aged 22-25 and 60-65 years, the D-A-CH values for the age groups 25-51 and >65 were used

[h]for the reference values of energy intake, the physical activity level is considered (range 1.4-1.8 PAL). In the present study, a medium physical activity level was assumed (PAL = 1.6). Since only a small proportion of the participants were aged 22-25 and 60-65 years, the values for the age groups 25-51 and >65 were used

[i]since there is no D-A-CH reference value, the EFSA (European Food Safety Authority) value was used

*Iodine without iodine from iodised salt (the German Food Code version 3.01 did not include iodised salt)
