# Supplementary material for: Dietary Nutrient Adequacy and Supplement Use in German Community-Dwelling Older Adults Compared to a Group of Younger People: Findings from the Berlin Aging Study II (BASE-II)
Source: Nutrients. 2026 Sep 20;18(18):3077. doi: 10.3390/nu18183077 (PMC13610988; doi:10.3390/nu18183077)
Supplement: Supplementary file 1 [file nutrients-18-03077-s001.zip › Table S2.pdf]

Table S2: Intake of macronutrients as particpants (%) with intakes >/< the respective Reference Value/Reference Value for maximum intake or Average Requirement (for protein).

		Age group 22-36 (n=490)	Age group 60-84 (n=1582)
**Nutrient**	**Reference Value (RV)[a]/Reference Value for maximum intake (RV_{MI})**	**% > RV/RV_{MI}**	**% > RV/RV_{MI}**
**Salt**	RV_{MI}		
All participants	< 6 g/day	36	37
Women (n=260/793)	< 6 g/day	15	18
Men (n=230/789)	< 6 g/day	61	55
**Alcohol**	RV_{MI}		
All participants	< 10 g/day[h]	38	44
Women	< 10 g/day	32	31
Men	< 20 g/ day	23	36
**SFA[b,c]**	RV_{MI}		
All participants	< (7-)10 En%	(99-)97	(100-)99
Women	< (7-)10 En%	(99-)97	(100-)99
Men	< (7-)10 En%	(100-)97	(100-)99
**PUFA[d,c]**	RV_{MI}		
All participants	up to 10 En%	3	4
Women	up to 10 En%	2	3
Men	up to 10 En%	4	5
**Total fat[c]**	RV		
All participants	< 30 En%	95	98
Women	< 30 En%	95	98
Men	< 30 En%	96	98
**Protein (2-fold RV)[e]**	RV_{MI}		
All participants	96 /114 g/day[k]	25	10
Women	96[i]/114 g/day[j]	4	3
Men	114[i]/134 g/day[j]	27	7
**Energy[f]**	RV		
All participants	2,100/1,900 kcal/d[k]	54	64
Women	2,100[i]/1,900 kcal/d[j]	33	47
Men	2,700[i]/2,500 kcal/d[j]	47	48
**Nutrient**	**Reference Value (RV)[a]/Average Requirement (AR)**	**% < RV/AR**	**% < RV/AR**
**EPA+DHA (ω-3)**	RV		
All participants	250 mg/ day	53	29
Women	250 mg/ day	64	37
Men	250 mg/ day	40	21
**Dietary fibre**	RV		
All participants	≥ 30 g/day	87	84
Women	≥ 30 g/day	93	89
Men	≥ 30 g/day	80	79
**Carbohydrates**	RV		
All participants	> 50 En%	89	95
Women	> 50 En%	87	93
Men	> 50 En%	92	98

**Protein**	AR[l]		
All participants	39.6 /37.7 g/day[k]	3.5	1.5
Women	39.6[g,i]/37.7 g/day[g,j]	5.4	2.5
Men	46.2[g,i]/44.1 g/day[g,j]	2.6	1.1

Interquartile Range: 25th to 75th percentile; SFA: Saturated fatty acids; PUFA: Polyunsaturated fatty acids; EPA+DHA: sum of eicosapentaenoic acid and docosahexaenoic acid; En%: percent of energy intake
[a] D-A-CH RV (for protein the EFSA RV_{MI}/AR were used due to lack of a D-A-CH value; for EPA+DHA the EFSA RV was used due to lack of a D-A-CH value),
[b] saturated fatty acids
[c] the median is reported as En%
[d] poly-unsaturated fatty acids
[e] EFSA value (in absence of an UL for protein, the EFSA considers protein intakes twice as high as the RV as safe for healthy adults [1])
[f] physical activity level (PAL) of 1.6
[g] EFSA value (Average Requirement/AR, given in kg/bodyweight/day, calculated in the present study by using the D-A-CH reference body weight for age groups 25-51 (60.0 kg for women and 70.7 kg for men) and ≥65 years (57.1 kg for women and 66.8 kg for men)[2])
[h]since the GV_{MI} was only derived for gender groups, in this analysis the lower value was chosen
[i]value for age group 22-36
[j]value for age group 60-84
[k]since there are no values for age groups, the lower value of the gender values was chosen for the analysis
[l]AR by EFSA [3]

1. EFSA (European Food Safety Authority). Scientific Opinion on Dietary Reference Values for protein. EFSA Journal 2012, 10, 2557, 66.
2. Energie. Referenzmaße von Körpergröße und Körpergewicht für Säuglinge, Kinder, Jugendliche und Erwachsene (Energy. Reference values for height and weight for infants, children, adolescents, and adults.). Available online: https://www.dge.de/wissenschaft/referenzwerte/energie/ (accessed on 10 October 2024).
3. EFSA (European Food Safety Authority). Dietary Reference Values for nutrients: Summary report. EFSA Supporting Publications 2017, 14, e15121, 92.
