# Supplementary material for: Dietary Nutrient Adequacy and Supplement Use in German Community-Dwelling Older Adults Compared to a Group of Younger People: Findings from the Berlin Aging Study II (BASE-II)
Source: Nutrients. 2026 Sep 20;18(18):3077. doi: 10.3390/nu18183077 (PMC13610988; doi:10.3390/nu18183077)
Supplement: Supplementary file 1 [file nutrients-18-03077-s001.zip › Table S3.pdf]

Table S3: Percentage of participants above the respective UL.

**Nutrient**	**UL (EFSA)**	**22-36 y Women<br>>UL (%)**	**22-36 y Men<br>>UL (%)**	**60-84 y Women<br>>UL (%)**	**60-84 y Men<br>>UL (%)**
Vitamin A (retinol+retinyl esters)	3000 µg RE/d	0	2.6	1.6	3.3
Copper	5 mg/d	0	2.0	0.1	1.0
Zinc	25 mg/d	0	0.9	0	0.8
