# Supplementary material for: Dietary Nutrient Adequacy and Supplement Use in German Community-Dwelling Older Adults Compared to a Group of Younger People: Findings from the Berlin Aging Study II (BASE-II)
Source: Nutrients. 2026 Sep 20;18(18):3077. doi: 10.3390/nu18183077 (PMC13610988; doi:10.3390/nu18183077)
Supplement: Supplementary file 1 [file nutrients-18-03077-s001.zip › Table S4.pdf]

Table S4: Percentage of the participants with vitamin and mineral intakes < AR*/EAR#.

| Nutrient | AR*/ EAR# | % <(E)AR | Nutrient | AR*/ EAR# | % <(E)AR |
|---|---|---|---|---|---|
| **Calcium (mg/d)*** | | | **Vitamin B1 (mg/d)#** | | |
| 22-36 y All *(n=490)* | 750 [a] | 26 | 22-36 y All | 0.9[i] | 25 |
| Women *(n=260)* | 750[a] | 35 | Women | 0.9 | 40 |
| Men *(n=230)* | 750[a] | 16 | Men | 1.0 | 13 |
| 60-84 y All *(n=1582)* | 750 | 34 | 60-84 y All | 0.9[i] | 22 |
| Women *(n=793)* | 750 | 38 | Women | 0.9 | 34 |
| Men *(n=789)* | 750 | 30 | Men | 1.0 | 15 |
| **Magnesium (mg/d)#** | | | **Vitamin B12 (µg/d)#** | | |
| 22-36 y All | 265[b,i] | 17 | 22-36 y All | 2.0 | 3.7 |
| Women | 265[b] | 24 | Women | 2.0 | 5.8 |
| Men | 350[b] | 33 | Men | 2.0 | 1.3 |
| 60-84 y All | 265[i] | 16 | 60-84 y All | 2.0 | 0.6 |
| Women | 265 | 23 | Women | 2.0 | 0.8 |
| Men | 350 | 35 | Men | 2.0 | 0.4 |
| **Copper (mg/d)#** | | | **Vitamin B2 (mg/d)*** | | |
| 22-36 y All | 0.7 | 0 | 22-36 y All | 1.3 | 32 |
| Women | 0.7 | 0 | Women | 1.3 | 49 |
| Men | 0.7 | 0 | Men | 1.3 | 14 |
| 60-84 y All | 0.7 | 0 | 60-84 y All | 1.3 | 26 |
| Women | 0.7 | 0 | Women | 1.3 | 35 |
| Men | 0.7 | 0 | Men | 1.3 | 16 |
| **Zinc (mg/d)*** | | | **Vitamin B3[c] (mg/d)#** | | |
| 22-36 y All | 7.6[i] | 6 | 22-36 y All | 11[i] | 0.6 |
| Women | 7.6 | 10 | Women | 11 | 1.2 |
| Men | 9.3 | 8 | Men | 12 | 0 |
| 60-84 y All | 7.6[i] | 8 | 60-84 y All | 11[i] | 0.2 |
| Women | 7.6 | 12 | Women | 11 | 0.3 |
| Men | 9.3 | 15 | Men | 12 | 0.1 |
| **Iodine (µg/d)#** | | | **Vitamin B6 (mg/d)*** | | |
| 22-36 y All | 95 | 8 | 22-36 y All | 1.3[i] | 30 |
| Women | 95 | 10 | Women | 1.3 | 48 |
| Men | 95 | 7 | Men | 1.5 | 22 |
| 60-84 y All | 95 | 12 | 60-84 y All | 1.3[i] | 20 |
| Women | 95 | 13 | Women | 1.3 | 31 |
| Men | 95 | 10 | Men | 1.5 | 21 |
| **Phosphorus (mg/d)#** | | | **Vitamin B9[d] (µg/d)*** | | |
| 22-36 y All | 580 | 0.6 | 22-36 y All | 250 | 64 |
| Women | 580 | 1.2 | Women | 250 | 72 |
| Men | 580 | 0 | Men | 250 | 54 |
| 60-84 y All | 580 | 0.3 | 60-84 y All | 250 | 55 |
| Women | 580 | 0.4 | Women | 250 | 66 |
| Men | 580 | 0.1 | Men | 250 | 44 |
| **Iron (mg/d)*** assuming a moderate absorption of 10 %[f] | | | **Vitamin C (mg/d)*** | | |
| 22-36 y All | 9.6[i] | 7 | 22-36 y All | 80[i] | 12 |
| Women | (11.2) | (37)[g] | Women | 80 | 15 |
| Men | 9.6 | 7 | Men | 90 | 15 |

| | | | | | |
|---|---|---|---|---|---|
| 60-84 y All | 9.6 | 10 | 60-84 y All | 80[i] | 7 |
| Women | 9.6 | 15 | Women | 80 | 7 |
| Men | 9.6 | 5 | Men | 90 | 13 |
| **Iron (mg/d)*** assuming a high absorption of 16/18 %[h] | | | **Vitamin D (µg/d)[#]** | | |
| 22-36 y All | 6[i] | 0 | 22-36 y All | 10 | 95 |
| Women | (7) | (2)[g] | Women | 10 | 99 |
| Men | 6 | 0 | Men | 10 | 91 |
| 60-84 y All | 6 | 0.3 | 60-84 y All | 10 | 93 |
| Women | 6 | 0.3 | Women | 10 | 99 |
| Men | 6 | 0.3 | Men | 10 | 87 |
| **Vitamin A[e] (mg/d)** | | | | | |
| 22-36 y All | 0.49[i] | 0.6 | | | |
| Women | 0.49 | 1.2 | | | |
| Men | 0.57 | 0.4 | | | |
| 60-84 y All | 0.49[i] | 0.1 | | | |
| Women | 0.49 | 0 | | | |
| Men | 0.57 | 0.6 | | | |

*AR (Average Requirement) by EFSA; [#]EAR (Estimated Average Requirement) by IOM
[a]the AR for the age group 18-24 years is 860 mg/d; since only a small percentage of this study´s age group 22-36 years was of the ages 22-24, the AR of 750 was chosen
[b]the EAR for the age group 18-24 years is 330 and 255 mg/d; since only a small percentage of this study´s age group 22-36 years was of the ages 22-24, the EAR of 350 and 265 were chosen
[c]Niacin equivalents
[d]Folate equivalents
[e]Retinol equivalents
[f]according to Allen et al. [1]
[g]these data are only demonstrated for comparison purposes with the results of the probabilistic approach
[h]these absorption rates were used by EFSA to calculate the AR (16 % for men and 18 % for women)
[i]since the (E)AR were only derived for gender groups, in this analysis the respective lower value was chosen for the assessment in the age groups; younger women were excluded from the iron analysis

1. Allen, L.H.; Carriquiry, A.L.; Murphy, S.P. Perspective: Proposed Harmonized Nutrient Reference Values for Populations. Adv Nutr 2020, 11, 469-83.
