# Supplementary material for: Dietary Nutrient Adequacy and Supplement Use in German Community-Dwelling Older Adults Compared to a Group of Younger People: Findings from the Berlin Aging Study II (BASE-II)
Source: Nutrients. 2026 Sep 20;18(18):3077. doi: 10.3390/nu18183077 (PMC13610988; doi:10.3390/nu18183077)
Supplement: Supplementary file 1 [file nutrients-18-03077-s001.zip › Table S5.pdf]

Table S5: Highest Prevalences of intakes <(E)AR/RV and >UL/RV in macronutrients and micronutrients.

Nutrient	Age group/Sex	Prevalence of Intake <(E)AR[a]/RV[b]	Prevalence of Intake >UL/RV[c]
Calcium	Older women	38 %	
Magnesium	Older men	35 %	
Iron	Younger women	37-58 % (probabilistic approach)	
Copper	Younger men		2.0 %
Zinc	Younger men		0.9 %
Vitamin A (sum of retinol/retinylesters)	Older men		3.3 %
Vitamin B1 (Thiamine)	Younger women	40 %	
Vitamin B2 (Riboflavine)	Younger women	49 %	
Vitamin B6 (Pyridoxine)	Younger women	48 %	
Vitamin B9 (Folate)	Younger women	72 %	
Vitamin D	Women	99 %	
Energy	Older men		48 % > RV
Saturated Fatty Acids (SFA)	Older men+women		99-100 % > RV
Total fat	Older men+women		98 % > RV
Alcohol	Older men		36 % > RV
Salt	Younger men		61 % > RV
Protein	Younger men		27 % > 2 x RV
Carbohydrates	Older men	98 %	
Dietary fibre	Younger women	93 %	

[a]shown for values above 15 % of the participants <(E)AR
[b]only for Dietary fibre and Carbohydrates
[c]for macronutrients
